# The oldest plans to scale of humanmade mega-structures

**DOI:** 10.1371/journal.pone.0277927

**Published:** 2023-05-17

**Authors:** Rémy Crassard, Wael Abu-Azizeh, Olivier Barge, Jacques Élie Brochier, Frank Preusser, Hamida Seba, Abd Errahmane Kiouche, Emmanuelle Régagnon, Juan Antonio Sánchez Priego, Thamer Almalki, Mohammad Tarawneh

**Affiliations:** 1 CNRS, Archéorient, UMR 5133, Maison de l’Orient et de la Méditerranée, Université Lyon 2, Lyon, France; 2 MEAE, CNRS, USR 3135, Institut Français du Proche-Orient (Ifpo), East-Jerusalem, Palestinian Territories; 3 CNRS, Minist Culture, LAMPEA, UMR 7269, MMSH, Aix Marseille Univ, Aix-en-Provence, France; 4 Institute of Earth and Environmental Sciences, University of Freiburg, Freiburg, Germany; 5 UCBL, CNRS, INSA Lyon, LIRIS, UMR5205, Université de Lyon, Villeurbanne, France; 6 Ministry of Culture, Heritage Commission, Riyadh Department, Riyadh, Saudi Arabia; 7 Petra College for Tourism and Archaeology, Al-Hussein Bin Talal University, Wadi Musa, Jordan; Universita degli Studi di Milano, ITALY

## Abstract

Data on how Stone Age communities conceived domestic and utilitarian structures are limited to a few examples of schematic and non-accurate representations of various-sized built spaces. Here, we report the exceptional discovery of the up-to-now oldest realistic plans that have been engraved on stones. These engravings from Jordan and Saudi Arabia depict ‘desert kites’, humanmade archaeological mega-traps that are dated to at least 9,000 years ago for the oldest. The extreme precision of these engravings is remarkable, representing gigantic neighboring Neolithic stone structures, the whole design of which is impossible to grasp without seeing it from the air or without being their architect (or user, or builder). They reveal a widely underestimated mental mastery of space perception, hitherto never observed at this level of accuracy in such an early context. These representations shed new light on the evolution of human discernment of space, communication, and communal activities in ancient times.

## Introduction

### Discovery context

For at least 40,000 years, humans have reproduced mental images of their natural surroundings by sculpting objects and by painting and engraving long-lasting physical surfaces. In particular, designing plans and maps, or creating rational two-dimensional images of three-dimensional spaces at a reduced scale, was a momentous cognitive development in abstract thinking and representation [1]. However, while human constructions have modified natural spaces and their surroundings for many millennia, few plans or maps of such humanmade structures predate the protohistoric period of the literate civilizations of Mesopotamia and Ancient Egypt. Indeed, archaeological and historical research have only documented a few architectural plans and miniature models of buildings and large-sized objects from that time period (e.g.: domestic and ritual buildings, boats) [2, 3]. Before that, it is unknown how Stone Age communities conceived their buildings and use of their domestic or utilitarian structures, although a few examples show schematic, non-accurate, representations of various-sized built spaces [4, 5].

For the specific case of ‘desert kites’, which are prehistoric stone structures used as mega-sized traps to hunt wild animals, the existence of such representations is of the utmost importance for understanding how they were conceived and perceived in the landscape, at a time when mapping was unknown [6, 7]. We report here the exceptional discovery of the up-to-now oldest realistic plans, engraved on stones, of some of these humanmade archaeological mega-traps, from south-eastern Jordan and northern Saudi Arabia, the oldest of which are dated to 9,000 years ago.

Unlike other evidence for generic and rough representations of such structures, these engravings are extremely precise depictions of neighboring desert kite structures dated to the Neolithic [7, 8]. Such depictions were necessarily designed by the constructors and/or the users of the desert kites themselves, as the whole structure layout is impossible to grasp without seeing it from the air or without being their creator. They reveal a widely underestimated mental mastery of large structures, human landscapes and social spaces, hitherto never observed at this level of accuracy in such an early chrono-cultural context. Such plans may have been used for enhancing collective hunting strategies with these mega-traps. These representations shed new light on the evolution of human perception of space and communal activities in ancient times, and highlight proto-forms of inscribed communication, going beyond the mere depiction of a mental representation of a large-scaled structure.

### Desert kites: Definition and general issues

**Fig 1:**
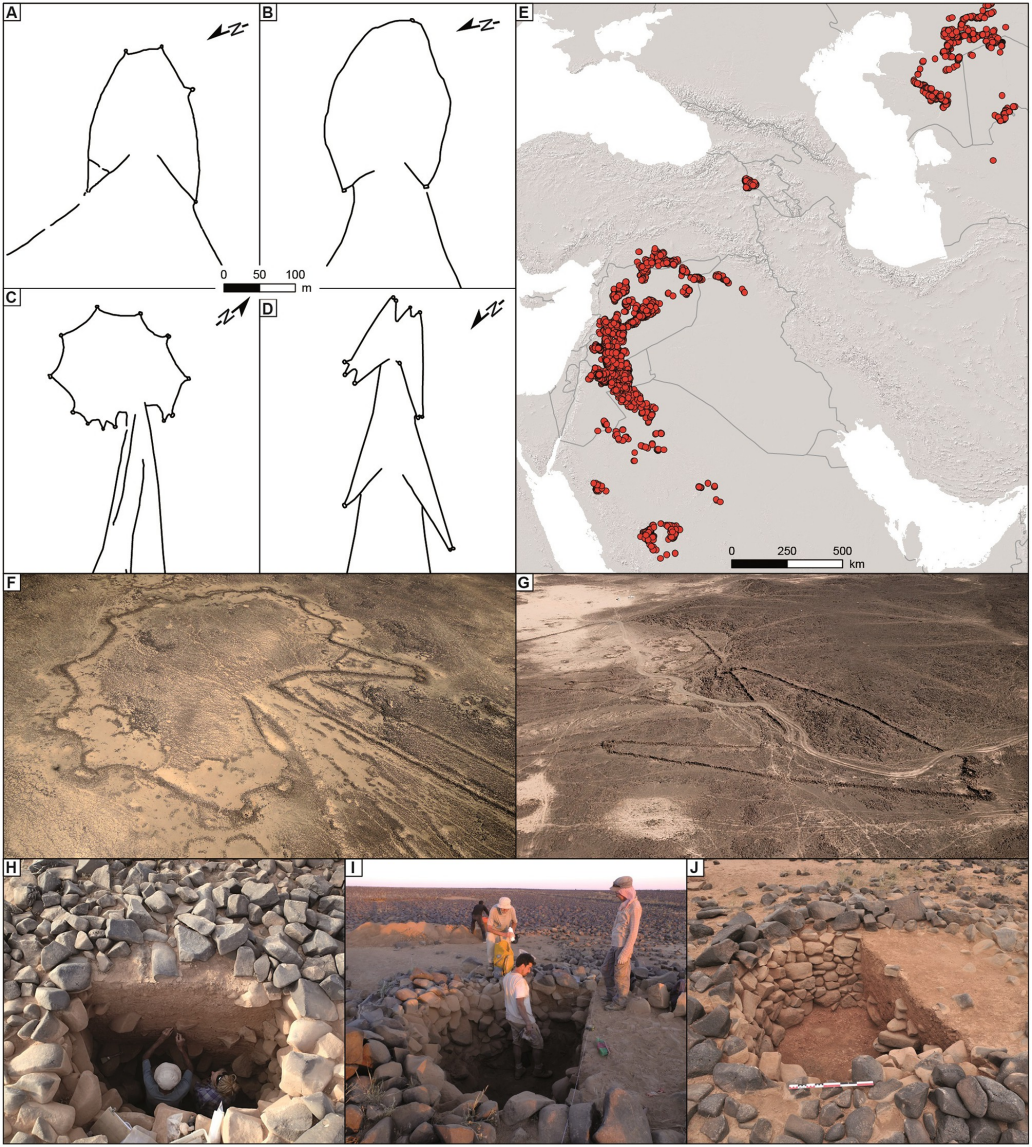
Distribution and characterization of desert kites. (A) Desert kite plan from Kazakhstan (Ustyurt Plateau). (B) Desert kite plan from Armenia (Mount Aragats). (C) Desert kite plan from Jordan (Harrat al-Shaam). (D) Desert kite plan from Saudi Arabia (Khaybar). (E) Distribution area of desert kites from western Arabia to Uzbekistan. (F) Oblique aerial picture of a desert kite in Jordan (Harrat al-Shaam, photo OB, Globalkites Project). (G) Oblique aerial picture of a desert kite in Saudi Arabia (Khaybar, photo Khaybar Longue Durée Archaeological Project, RCU-AFALULA-CNRS). (H), (I), (J) Desert kite pit-traps during and after excavation (of half the pit) in the Harrat al-Shaam region of Jordan (photos RC, OB, WAA, Globalkites Project).

Desert kites (or simply kites) are gigantic archaeological structures made of stone alignments and walls. Kites are composed of driving lines (from hundreds of meters to 5 km long) converging towards an enclosure (median size: 1 ha), which is surrounded by up to 4-meter-deep pits (called ‘pit-traps’, from 1 to more than 20 in number per enclosure) where animals were trapped by hunters [7] (Fig 1, and S1–S4 Figs). They represent some of the most impressive stone-built constructions erected by humans in recent prehistory. Desert kites are the earliest large-scale monuments known to date, dating back to as early as 9,000 years ago, during the Pre-Pottery Neolithic B (PPNB) period in Jordan [8]. Elsewhere, some of them were in use in more recent times [9].

Hunting in past societies has been widely studied by archaeologists for decades but hunting using trapping techniques has rarely been comprehensively addressed. Our recent studies of kites in various regions propose to bridge this gap by applying a multi-proxy approach to the worldwide expansion of these spectacular types of traps, drawing on diverse disciplines such as archaeology and social anthropology, bioarchaeology and geoarchaeology, geomatics and statistics, and geochronology [6]. The whole dataset is based on a large body of archaeological structures initially detected from satellite imagery, and then studied in the field. These massive structures visible from airplanes were first recognized in the 1920s and were quickly interpreted as hunting traps, which was confirmed by recent archaeological excavations [6, 7, 10–12]. Until recently, almost no in-depth studies had been carried out to enhance our understanding of their function, functioning, chronology or why they were so widespread in many regions ranging from Arabia to Uzbekistan. Kites are highly sophisticated structures in the landscape involving mass hunting strategies of wild animal herds, far from settled areas, in what are today arid environments. They reflect the emergence of innovative strategies of animal resource procurement (essentially meat, but most likely other resources such as horns, hair and hide), extending beyond the sole purposes of subsistence. This new adaptive choice, inducing the artificialization of landscapes and the probable progressive degradation of biodiversity (materialized by possible extinctions of animal species) started at a time when agriculture and domesticate farming was established in neighboring regions of the Near East by well-settled societies.

### Regional setting and location of the engravings

Currently, 6,255 desert kites have been recorded in the kite distribution area across the Middle East, Caucasia, and Central Asia [6, 7, 9, 13–17] (for permanently updated information, notably on the total number of kites, see: www.globalkites.fr). The lava fields known as Harrat al-Shaam in southern Syria, eastern Jordan and northern Saudi Arabia, contain the most numerous and the highest density of kites (up to one kite/sq. km). Two specific zones of investigation at the edge of the lava fields, with different geographical and geological features, yielded two rock engravings depicting kites to scale. These are the Jibal al-Khashabiyeh plateau at the eastern edge of the Al-Jafr Basin in south-eastern Jordan and the Jebel az-Zilliyat plateau at the northern edge of the Nefud Desert in northern Saudi Arabia, separated by 267 km.

**Fig 2:**
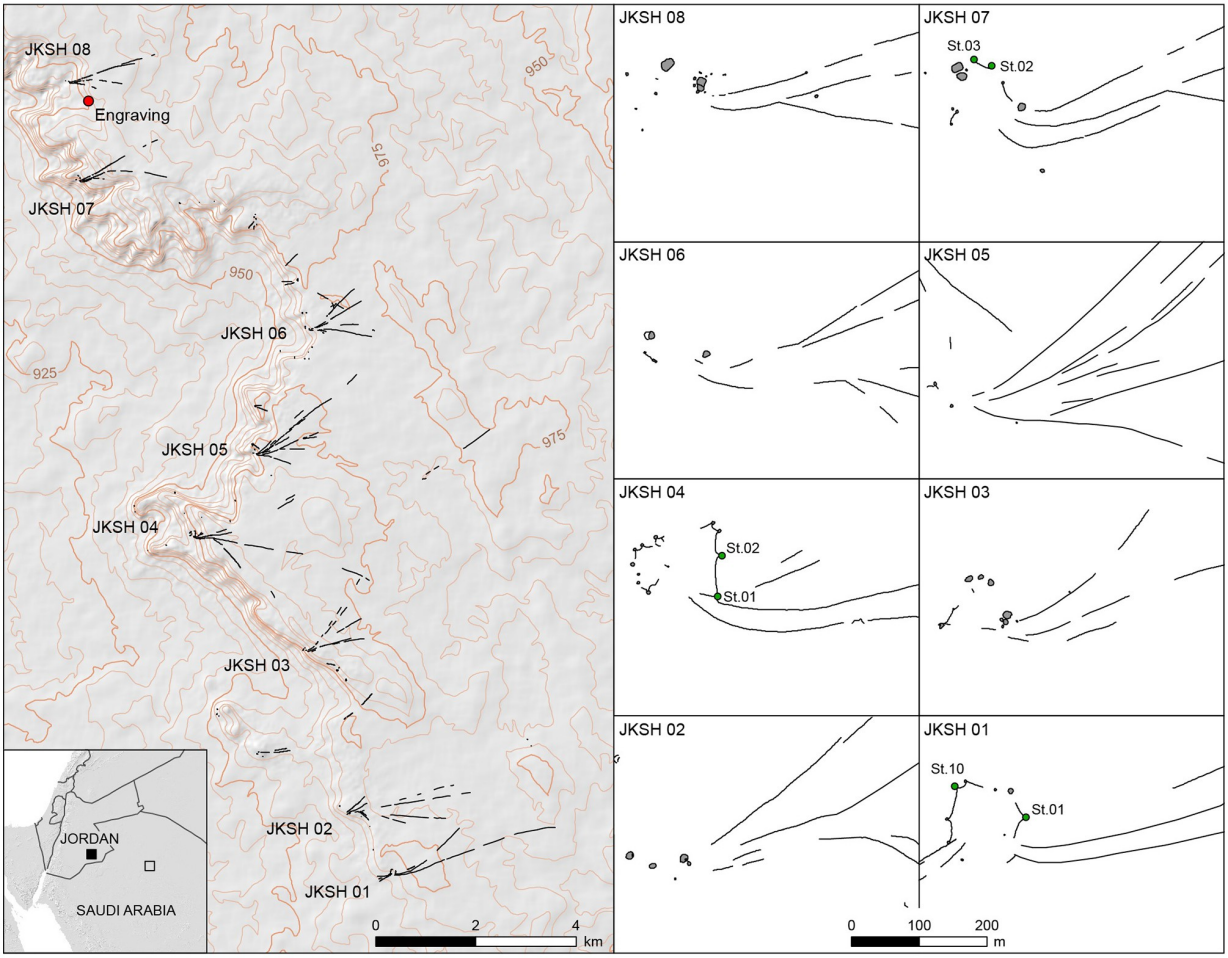
Location of fieldwork in Jordan. Location of the eight desert kites in the Jibal al-Khashabiyeh region in south-eastern Jordan (left), and detailed plans of these kites (right), with mentions (St.n) of excavated pit-traps. The red dot in the general map (left) shows the location of the engraving, found at the JKSH F15 site. The green dots in the kite plans indicate the excavated pit-traps and their numbering.

In Jordan, the Jibal al-Khashabiyeh area, located 80 km east of Al-Jafr city, is marked by a limestone plateau escarpment with a maximum elevation of 1,000 m above sea level, sloping to the east. A major hydrographic network dissects this escarpment towards the Al-Jafr basin. The limestone bedrock is rich in chert, particularly on the current surface of the desert rocky pavement and along the upper parts of the escarpment slope. The top of the escarpment yielded eight kites (S1 Table), built with the whitish limestone blocks and piles of chert slabs covering the surface of the desert, in order to visually mark the generally dark landscape [12] (Fig 2). The same number of archaeological complexes, interpreted as campsites, are associated with the kites. Three campsites have been partially excavated, including a rescue excavation at the looted site F15 (close to kite JKSH 08), where a stone engraved with a kite representation was found.

**Fig 3:**
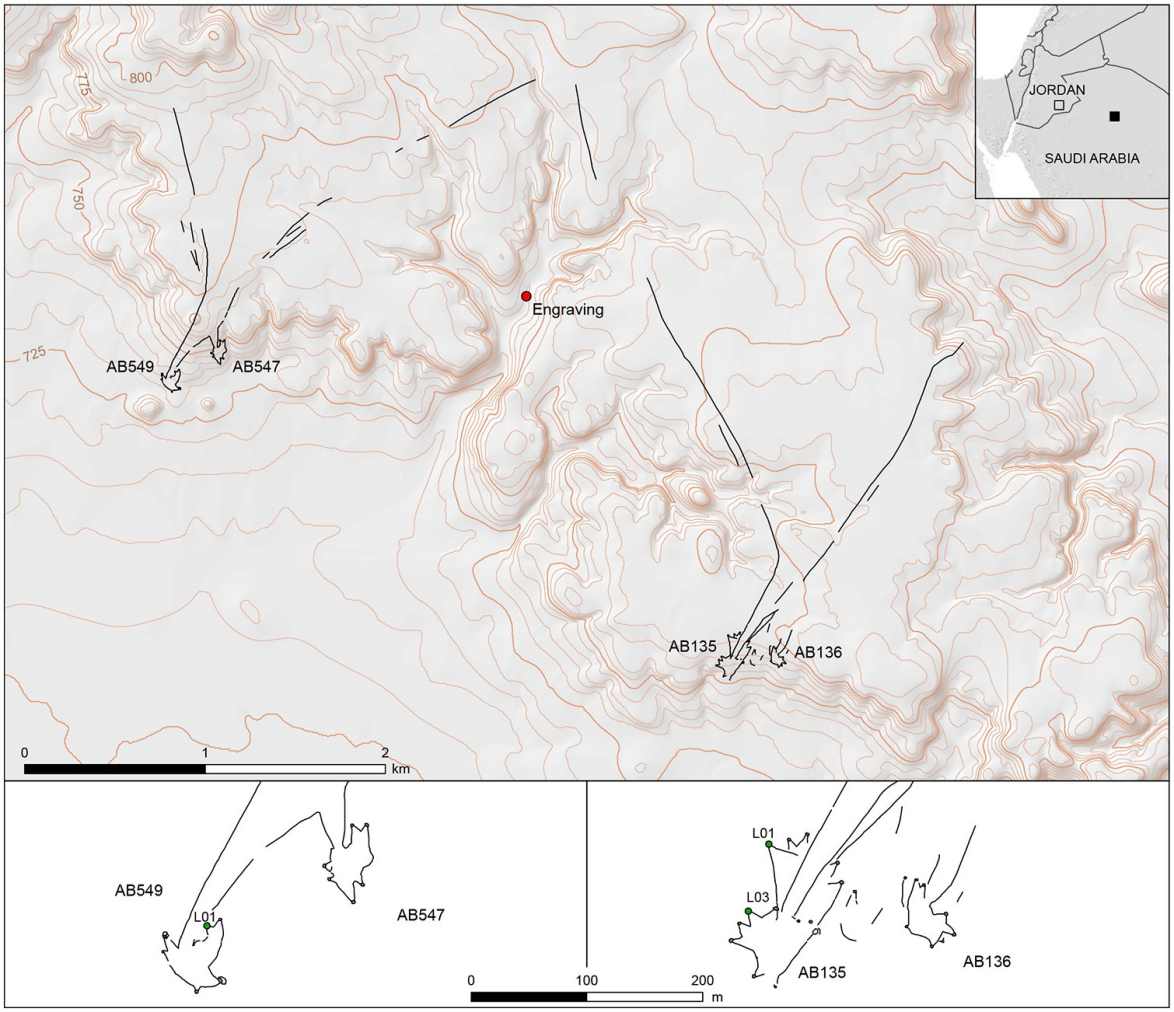
Location of the fieldwork in Saudi Arabia. Location of the four desert kites in the Jebel Az-Zilliyat region in northern Saudi Arabia (top), and detailed plans (bottom) of the two pairs of kites. The red dot in the general map (top) shows the location of the engraving, in the bed of Wadi az-Zilliyat. The green dots in the kite plans indicate the excavated pit-traps and their numbering.

Some 260 km to the east, Jebel az-Zilliyat in Saudi Arabia is part of a complex topography of the central part of the Al-Jawf province comprising numerous grabens and faults, including the Wadi As-Sirhan fault, which is one of the most significant geological features. A few kilometers to the north, the exposed bedrock mainly consists of sandstone, where the large and horizontal Jebel az-Zilliyat plateau is dissected by faults and drainage features. Two pairs of kites are visible (kites AB135/AB136 and AB547/AB549; S2 Table) on the edge of the plateau cliff, with a distance of 3.5 km separating the two distinct groups. A pedestrian survey in one of the drainage features of the plateau revealed a massive engraving to scale representing two kites, equidistant from these two pairs (Fig 3).

## Results

### The Jibal al-Khashabiyeh engraved stone

**Fig 4:**
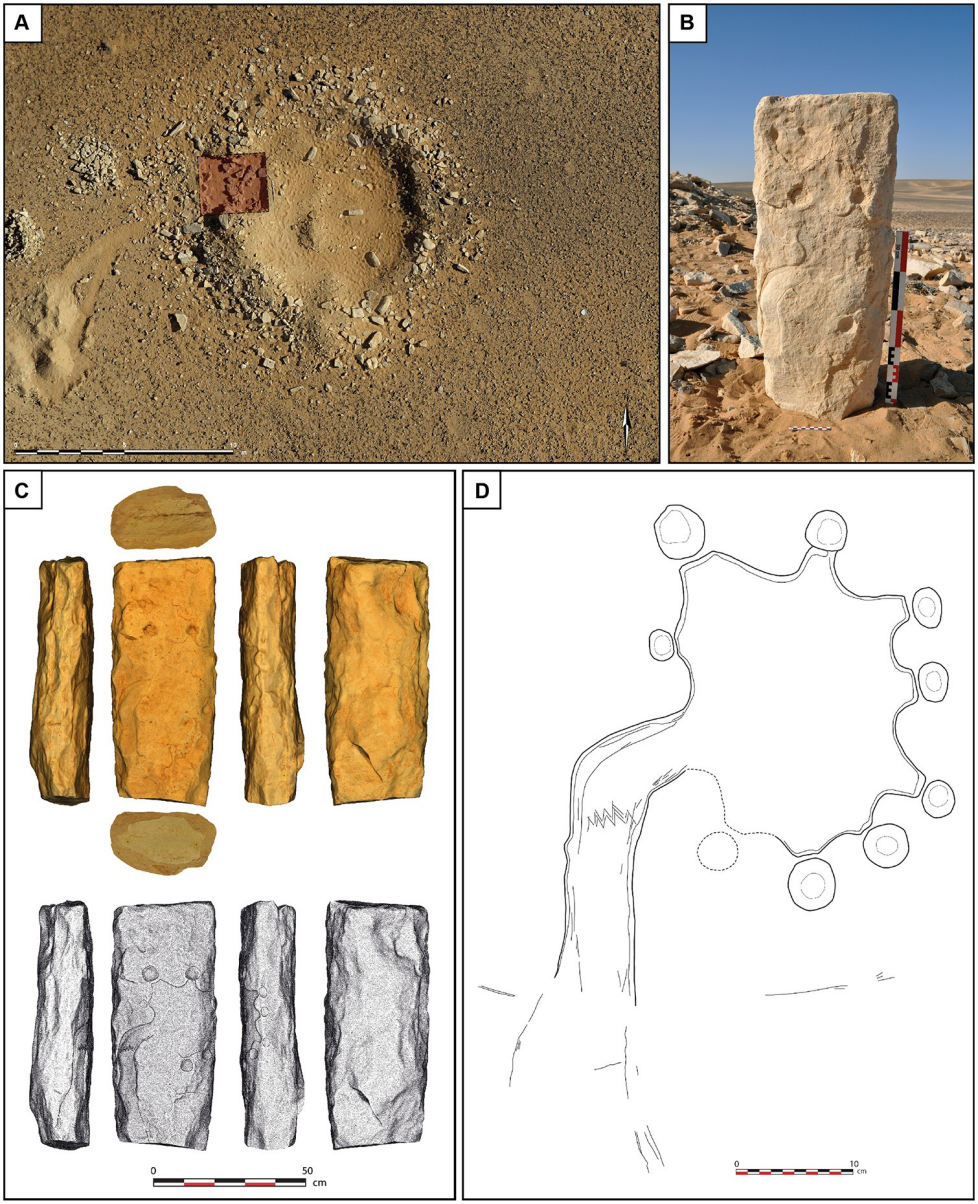
Discovery of the engraved stone in Jibal al-Khashabiyeh, Jordan. (A) Orthomosaic view of JKSH F15 site where the kite’s engraving was found on a monolith (in red is the location of the rescue excavation in the looter’s spoil). (B) Photograph of the engraved stone at the time of discovery at the JKSH F15 site (the monolith was found lying down and was set vertically for the photograph). (C) Photogrammetric 3D model of the engraved monolith showing the different faces, including the engraved upper face (top), while the hill-shaded model (bottom) shows the interpretative drawing of the engraved plan on the stone. (D) Drawing of a projected view of the kite representation engraved on the monolith from the JKSH F15 site.

At Jibal al-Khashabiyeh, the F15 site is a PPNB settlement disturbed by recent looting activities (Fig 4A). Architectural remains are nevertheless still visible on the current surface of the ground, including numerous carved stones scattered amongst the looters’ spoil. These comprise elements comparable in shape to those known as ‘cigar-shaped stones’ in the architectural typology of the Mureybetian culture of the Near Eastern Pre-Pottery Neolithic A (PPNA) [18, 19]. On one of these limestone monoliths, 80 cm-long, 32 cm-wide and 18 cm-thick (exact dimensions are: length between 78.27 and 82.76 cm; width between 29.18 and 34.67 cm; thickness between 17.11 and 19.22 cm), found on the surface of the site in June 2015 (Fig 4B), a very well-preserved engraving of a kite is clearly visible (Fig 4C and 4D). The weight of this heavy monolith is 92 kg.

**Fig 5:**
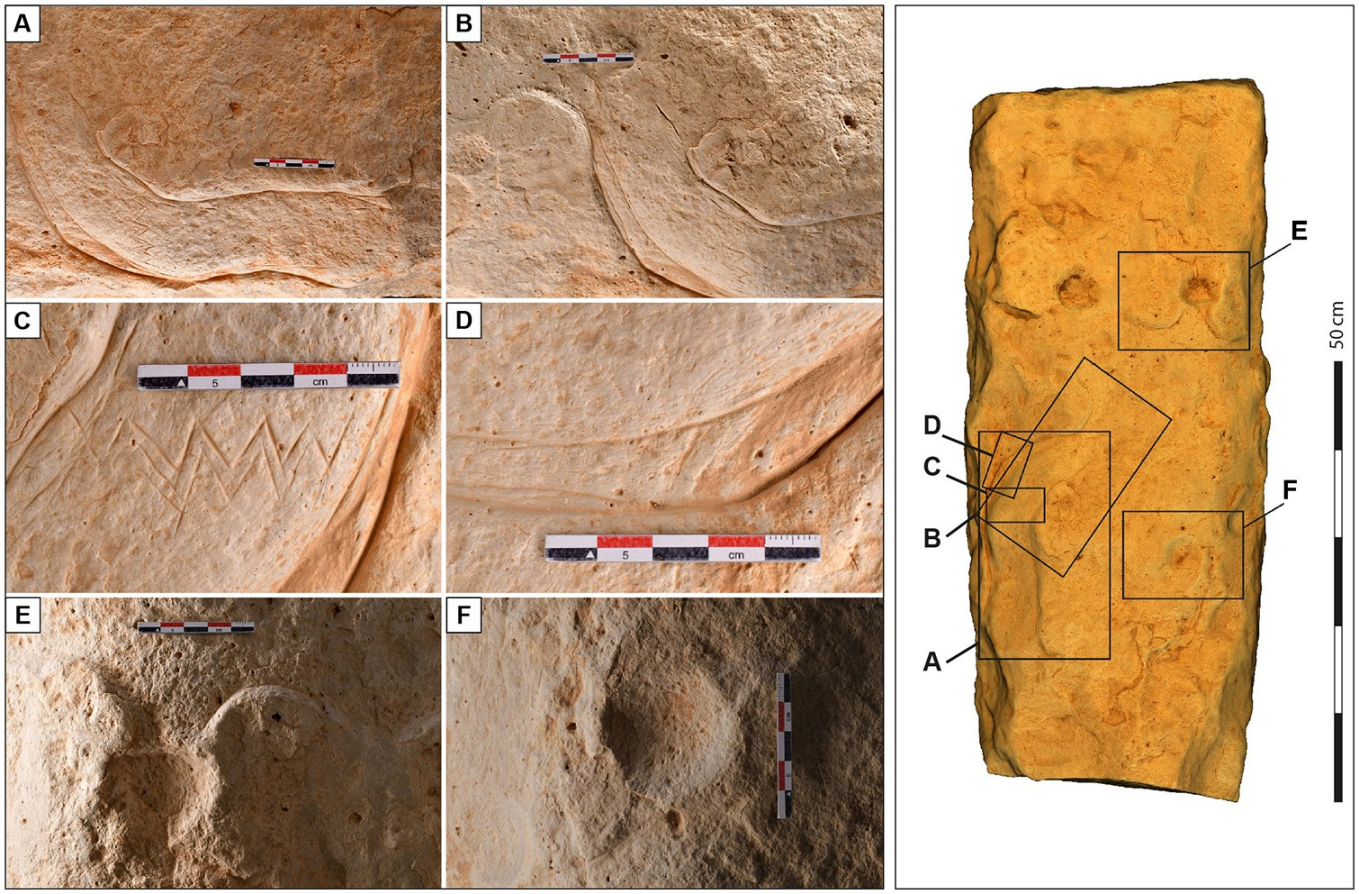
Detailed photographs of the engraved stone surface from Jibal al-Khashabiyeh, Jordan. The detailed views emphasize the various techniques used for the kite engraving found at the JKSH F15 site. The detailed photographs on the left are lettered, indicating their position on the engraved monolith, as shown on the right.

The edges of the limestone block were carved with a massive hard hammer. The engraving is made of a combination of multiple carving techniques, including fine incisions (notably to delineate the contours of the kite) and pecking (Fig 5 and S5 Fig). It was most probably produced with a lithic tool on rather chalky, easy to incise limestone, such as a burin, a scraper, or even a raw flake or blade. High-quality natural and human-transformed chert literally covers the area and could have been used for the task. Technically, the kite is depicted in low relief, as the entire internal space of the kite has been carved out and smoothed to a depth of ca. 0.5–1 cm. This could have been achieved using a more massive lithic tool such as a thick flake or an adze-like tool.

The engraving depicts a kite structure with its three essential components (Fig 4D). The driving lines are represented by two main converging curved lines of ca. 40 cm in length, leading to a carved star-shaped enclosure of approximately 26 cm in diameter, at the circumference of which are eight circular cupules (cup-marks), ranging from 2.5 to 6 cm in diameter, representing the pit-traps of the kite. To the right of the entrance of the enclosure, part of the kite plan is missing, either due to weathering and exfoliation of the monolith, or because the engraving was unfinished. Each one of these cupules is located at the extremity of the star points. Given the overall symmetry in the drawing layout, an additional cupule was probably intended to be drawn at this specific location, bringing the total number of circular pit-traps on the engraving to nine. The driving lines run almost parallel to each other, forming a narrow and elongated corridor, before turning at an almost 90° angle to the right, where they finally converge towards the narrow entrance of the star-shaped enclosure. This overall layout and organization–the narrow corridor funnel shape, and the marked curve immediately preceding the final convergence point at the entrance of the enclosure–constitute a local structural characteristic of neighboring south-eastern Jordanian kites. This plan has not been documented elsewhere in the kite concentration in north-eastern Jordan, and only rarely in the northern Saudi Arabian group. A comparable shape is found in Saudi Arabia, at distances of 85 km and 270 km for the two closest examples (AB754: 29.989N; 37.881E and AB557: 29.24N;39.554E).

Just before the curvature of the driving lines, a zigzag pattern of two to three incised parallel lines runs perpendicularly across the corridor width, forming a regular design of five chevrons. The exact meaning of this graphic pattern is unknown, but we postulate that it is of symbolic significance and could either indicate an unusual device–possibly in light materials and therefore not preserved–related to the kite’s functioning (such as a beater-driven hunting spot or a net), or a representation of a topographic slope break feature. In view of our understanding of the overall functioning of kites gleaned from excavation results and spatial analysis [7], the latter hypothesis seems more likely. Indeed, the presence of a specific device at this strategic location seems incongruous. On the other hand, the slope constitutes a conspicuous feature in the landscape and setting of kites, and is believed to be an active part of the successful functioning of kites [12]. The weathering and erosion of the harder upper layer of limestone evidences a fragmentation pattern of an irregular line along the escarpment slope, and the graphic chevron pattern could be a seemly schematic representation of this (S6 Fig).

### The Jebel az-Zilliyat engraved rock

**Fig 6:**
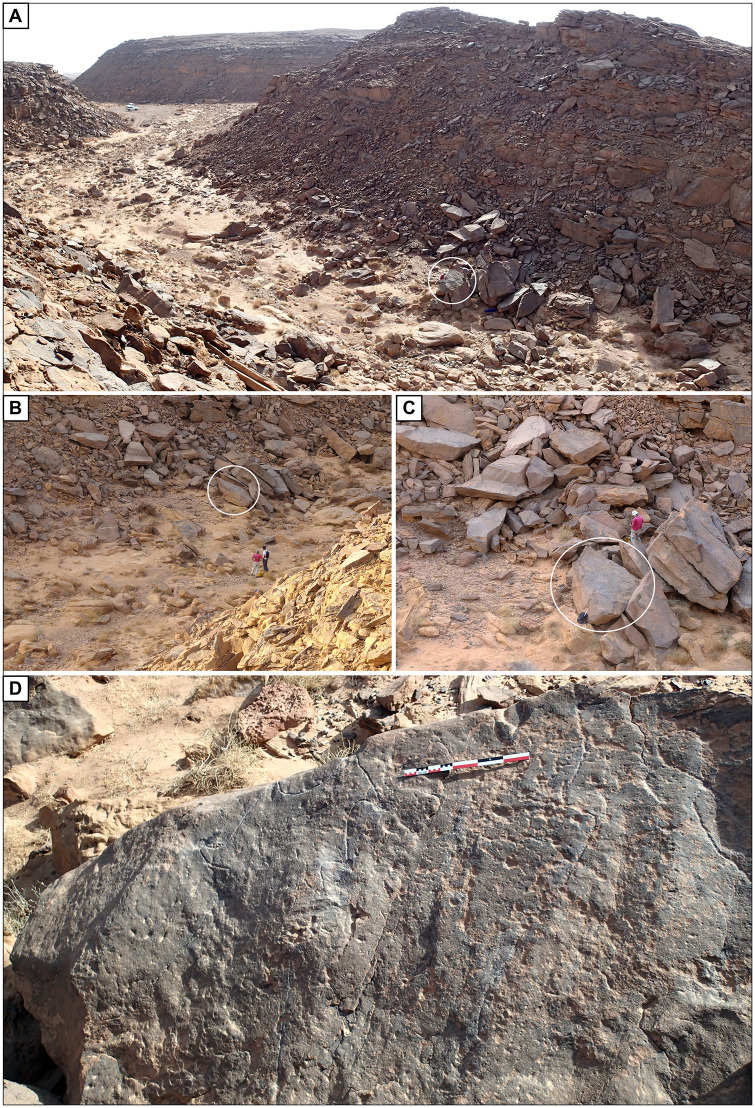
Location of the engraved rock in Wadi az-Zilliyat, Saudi Arabia. (A) General view of Wadi az-Zilliyat, from the northeast, the location of the engraved boulder is shown by the white circle. (B) General view of the collapsed boulders from the southeast, the white circle indicates the position of the engraving. (C) General view of the engraved rock (white circle) location among the collapsed boulders, from the east. (D) The engraved boulder as discovered during rock art survey, view from the north.

**Fig 7:**
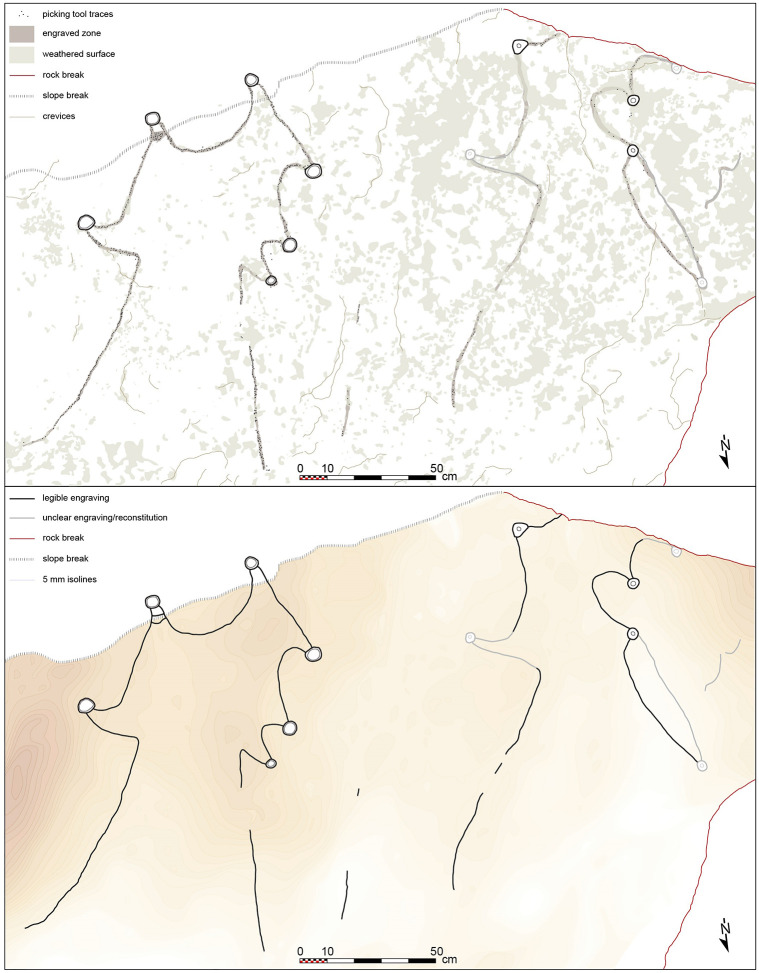
The engraved boulder from Jebel az-Zilliyat, Saudi Arabia. (A) Drawing of a projected view of the kites’ representation showing picking tool traces, engraved and weathered zones. (B) Drawing of a projected view of the kites’ representation showing legible and unclear engravings, with a colored restitution of the microtopography of the boulder surface.

**Fig 8:**
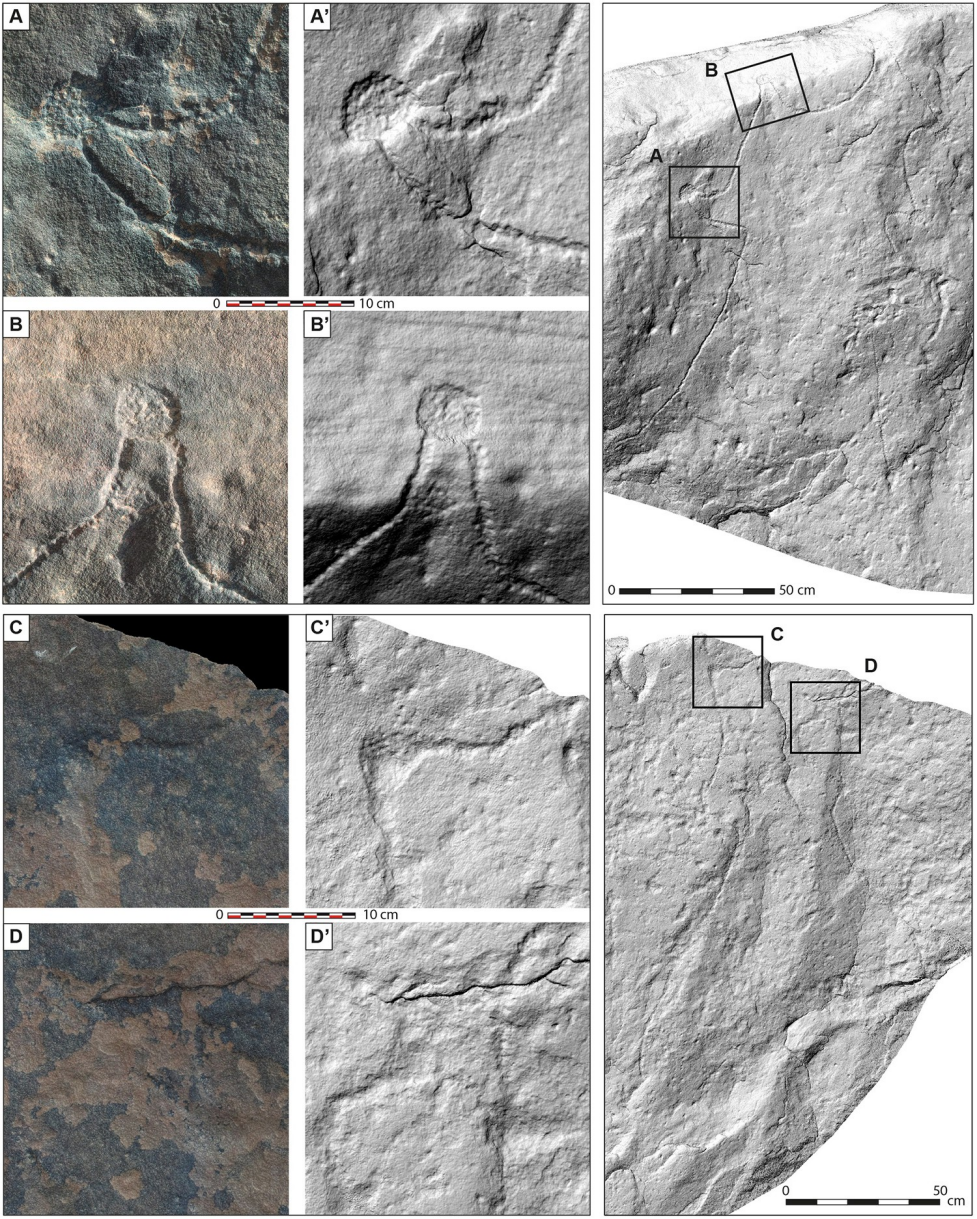
Detailed photographs and hill-shaded surface models of the engraved stone surface from Jebel az-Zilliyat, Saudi Arabia. The detailed views emphasize the various techniques used for the kite engraving. The detailed photographs and hill-shaded surface models on the left are lettered, indicating their position on the engraved boulder, as shown on the right. A and B are showing examples from the eastern depiction of a kite, C and D from the western one. Hill-shaded surface models are used to better render the legibility of the engravings, otherwise less visible from the photographs made on site.

The Jebel az-Zilliyat representation is much larger and was found during rock art surveys in March 2015 in Wadi az-Zilliyat cutting through Jebel az-Zilliyat (Fig 6). Two graphic units representing two kites are visible on a 382 cm-long and 235 cm-wide (maximum visible width) flat surface of a massive 105 cm-thick sandstone boulder, which fell to the wadi bed from the overhanging cliff edge (Fig 7). The kite representation on the eastern part of the boulder is almost entirely readable, while the one on the western part is extensively damaged by erosion. Unlike the Jibal al-Khashabiyeh example, the rock engravings at Jebel az-Zilliyat are exclusively made by pecking with unknown tools (Fig 8), possibly a lithic burin or a more massive hand-pick. Again, high-quality chert is widely available locally and may have been used for the task. The eastern engraving shows a kite with two short and widely spaced convergent driving lines of 80 and 85 cm in length leading to a star-shaped enclosed surface of about 50 to 60 cm in width. Six cupules (pecked cup-marks) between 3 and 5.5 cm in diameter are located at the extremities of point-shaped appendixes. The two southern cupules are located on another plane of the main rock surface, along the upper part of the southern side of the boulder. The eastern one of the latter shows a traced delimitation at the base of the point-shaped appendix (Fig 8B). This feature is known in the field on current kites as a locked-point pit-trap type. The western engraving is much more difficult to read, as the exfoliated surface of the sandstone prevents a clear interpretation of the design. The general shape seems to be quite similar to the eastern kite, with the remains of at least four cupules between 3.5 and 4.5 cm in diameter representing the pit-traps (Fig 7). Two 85- and 60-cm-long converging lines lead to a smaller enclosure of about 40 to 45 cm in width. The southern extremity of the enclosure is missing, due to boulder fracturing. It is still unknown if the engraving was made on the cliff itself before the boulder collapsed, or if it was made on the collapsed boulder in the wadi bed.

### Description and dating of Jibal al-Khashabiyeh desert kites near the engraving

The dating of the engraved rocks themselves or the sedimentary deposit from which the rocks were extracted was impossible. Therefore, we compared the designs of the representations to neighboring kites in both regions, in order to infer the age of the engraved rocks. Many similarities were observed. Additionally, in Jibal al-Khashabiyeh, a radiocarbon date was obtained from the F15 site itself (8016 ±29 BP, 6930 ± 80 cal. BC), where the engraving was found. The dated sample consists of charcoal collected in a disturbed context, during a rescue excavation in the looters’ spoil, but the material clearly originates from the original occupation. The extent of the latter is limited with no evidence for successive reuse throughout time, as evidenced by the homogeneity of the material culture at the surface of the site. The date turned out moreover to be consistent with the dating of nearby kites.

**Fig 9:**
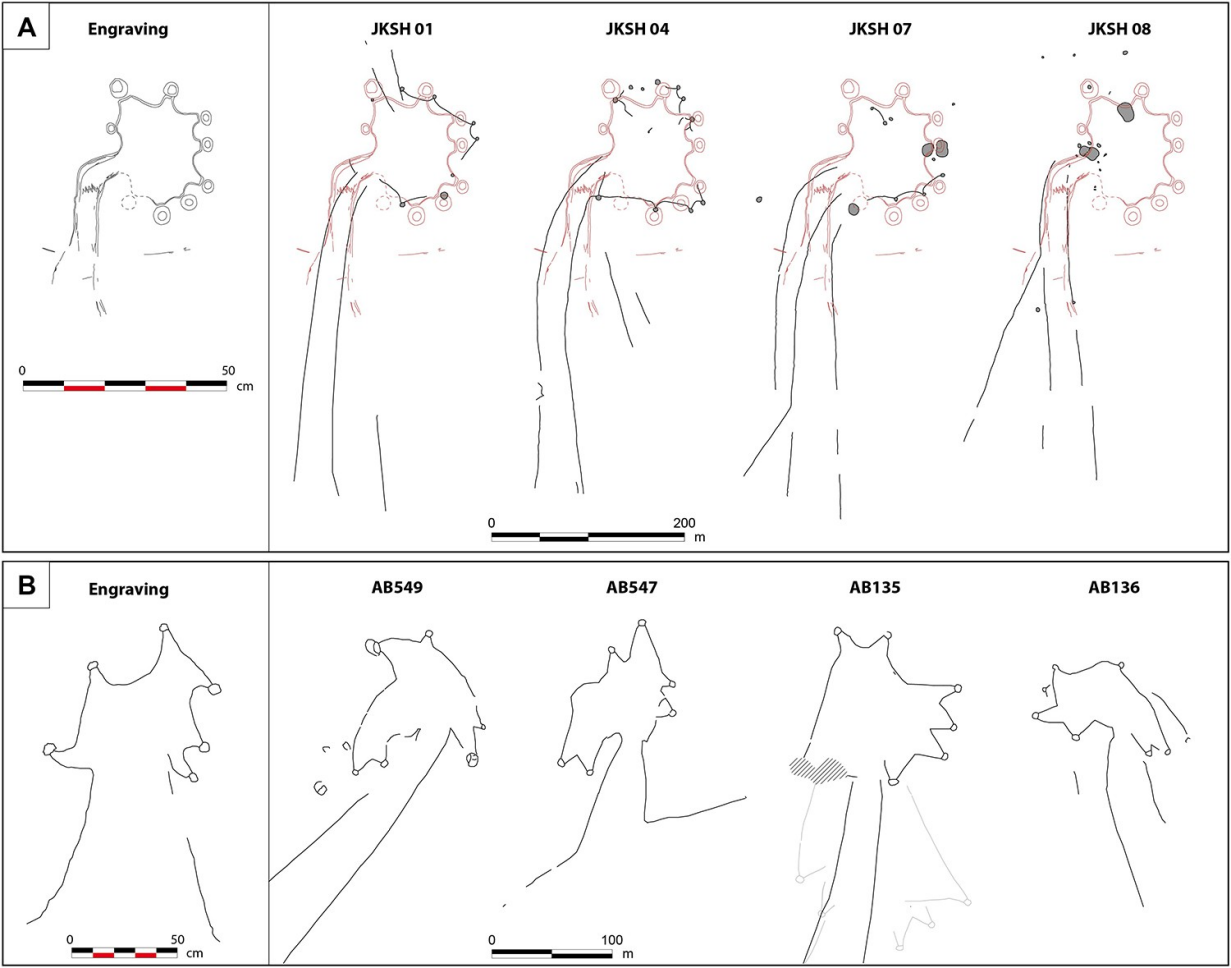
Comparison of the kite layouts depicted on the engravings with the top-view plans of neighboring desert kites in Jibal al-Khashabiyeh, Jordan and Jebel az-Zilliyat, Saudi Arabia. (A) Comparison of the kite layout depicted on the engraved monolith (left) with the top-view plans of the four better preserved kites identified in Jibal al-Khashabiyeh (right). The red dotted line is the shape of the kite engraving, used for superimposition on the desert kite maps. (B) Comparison of the kite engraving found at Jebel az-Zilliyat (left) with top-view plans of the four neighboring desert kites (right). Gray zones are destroyed or reused areas, after the period of kite use.

At Jibal al-Khashabiyeh, the general aspect of the eight kites is very similar to the engraving found at the F15 site. Among the eight kites, four display the characteristic shape of a narrow funnel formed by the driving lines before the marked curve preceding the final convergence point at the enclosure entrance (S7 Fig). Two of these kites, JKSH 07 and JKSH 08, are the nearest kites to site F15 where the kite engraving was discovered. These two kites show a specific pattern, with the southern driving line marking a sudden change in direction opening the funnel widely, while the northern driving line remains straight (Fig 9). A similar layout is depicted in the kite engraving (Fig 4D). As kite enclosures suffered heavy erosion due to their location at the starting point of intermittent water drainage systems, the star-shaped polygonal enclosures are partially preserved and only clearly identifiable at three kites (Fig 9A). At kite JKSH 01, eight pit-traps are still visible, and their original total number can be presumed to be nine (based on the regularity of the shape and estimated distance between each pit-trap; S7 Fig). At kite JKSH 04, eight are visible, and three additional pit-traps are probably missing, while at kite JKSH 07, only three are still visible and six additional pit-traps are hypothesized. The average number of pit-traps is therefore similar to the number of cup-marks on the kite engraving. These three kites were excavated. At each one of them, half or quarter of two peripheral pit-traps were excavated, revealing hollow structures with carefully built internal stone facing, and a depth ranging from 143 to 176 cm. Five radiocarbon datings on charcoal samples from the lower fill of the pit-traps at kite JKSH 01 provided an age of ca. 7,000 cal. BC (S3 Table) [8].

### Description and dating of Jebel az-Zilliyat desert kites near the engraving

At Jebel az-Zilliyat, the engraved rock is located in Wadi az-Zilliyat, between the western pair of kites AB547 and AB549 and the eastern pair AB135 and AB136 (Fig 9B). AB135 and AB136 are only 120 m apart, and their openings and funnel entrances are parallel to each other (Fig 3). As observed on the engravings, the orientation of the kites is similar, with the enclosure facing south (Fig 7). AB135 is a huge star-shaped example of a rather typical type of kite in the region. Three driving lines (3,379 m for the longest) lead to a 1.13 ha enclosure. The current state of preservation of the latter kite is very poor as many parts of the structure have been bulldozed by geophysical surveys for oil and gas exploration. The kite has also been partially reused by more recent (most probably later prehistoric) populations who built tombs (stone mounds, cairns) and circular structures (pastoral or dwelling enclosures) with stones from the kite. Nevertheless, field observations combined with aerial and satellite photo interpretation yield an accurate evaluation of the initial structural aspect of the AB135 kite. Standing tabular sandstone alignments and more rarely actual walls with two to three stone rows at most characterize the general construction technique. The AB135 kite presents four well-distinguished pit-traps on its north-western side, and two at its southern extremity (including the heavily damaged south-eastern one). These latter two are at the edge of the plateau’s cliff, and are intriguingly similar to the better-preserved kite engraving in the neighboring Wadi az-Zilliyat (S8 Fig). The eastern side of the kite is unfortunately too damaged and reused to gauge its original shape. Series of pit-traps are also visible at the northern part of the AB135 kite, along the driving lines, but construction techniques and desert varnish were slightly different from the main original kite star-shaped structure, and they are coarser in appearance. These field observations show that they were built at a later phase and are clearly add-ons. Two archaeological soundings were carried out in two of the peripheral pit-traps (AB135-L01 and AB135-L03). The sounding in the AB135-L01 pit-trap revealed very shallow infilling reduced to old red altered soil unsuitable for optically stimulated luminescence (OSL) or radiocarbon dating. The AB135-L03 pit-trap took advantage of the natural topography for the construction of its peripheral wall. The sounding in AB135-L03 yielded no artifacts but sediments yielded datable remains of five fragments of calcitic nutlets of *Arnebia* sp. (Boraginaceae) found about ten centimeters above the bottom of the pit-trap. This sample provided a conventional date of 6760 ± 40 BP (5670 ± 35 cal. BC). This age is confirmed by the OSL analysis dating of sediment samples from the lower part of the same pit-trap, which yielded a date of 7690 ± 400 years (5675 ± 400 BC). These dates give an age for the initial filling of the AB135-L03 pit-trap after the abandonment of the kite, by this indicating a minimum age for its construction (S4 Table).

The AB136 kite is less disturbed and damaged than its neighbor AB135 but consists of a right driving line 156 m in length, a left one of 54 m in length, an enclosure of 0.42 ha and six pit-traps. The driving lines might have worked together with those from AB135. The AB136 kite presented no pit-traps with enough sediment accumulation due to an unfavorable overall topographic context for good preservation. Thus, no excavations were carried out there. The western pair of kites AB547 and AB549, displaying the same construction techniques, is less disturbed and damaged. Two of their driving lines seem to have worked together (1,549 m and 2,623 m in length), while their enclosures respectively measure 0.55 and 0.63 ha, both with six peripheral pit-traps (Fig 3). At kite AB547, no pit-trap was considered suitable for excavation. All were either on a slope, or contained very little accumulated sediment, jeopardizing a secure context for geoarchaeological analyses or OSL sampling. The kite was accurately mapped using Differential GPS, showing its direct relation with the neighboring AB549 kite, and reinforcing field observations that seem to indicate that both kites functioned together simultaneously. At kite AB549, one cell was excavated (AB549-L01), with a maximum depth of 60 cm between the present-day surface and the bedrock. Vertical slabs were aligned for the construction of the inner pit wall, sometimes forming a double facing wall, and with four to five courses of flat horizontal stones. A few larger slabs were positioned over the top of this wall as a corbelled construction. No artifacts were found, but sediment sampling yielded 11 small fragments of *Arnebia* sp. nutlets (from a depth of 49 cm), providing a radiocarbon date of 3300 ±40 BP (1570 ± 50 cal. BC), while the OSL dating of sediment samples from the lower parts of the same pit-trap provided two ages of 8040 ± 430 years (6025 ± 430 BC) and 7480 ± 460 years (5465 ± 460 BC). The disagreement between the estimate from radiocarbon dating and those from OSL underlines the need to use jointly, when possible, different dating methods. In the present case, the potential mobility of the small fragments of *Arnebia* nutlets in the stratigraphy and the very small amount of carbon obtained after purification (0.2 mg) lead us to favor the results of OSL dating. These OSL dates give an age for the initial filling of the AB 549 LO1 pit-trap after the abandonment of the kite, by this indicating a minimum age for its construction (S4 Table).

### Quantitative comparison of the engravings and neighboring kites

**Fig 10:**
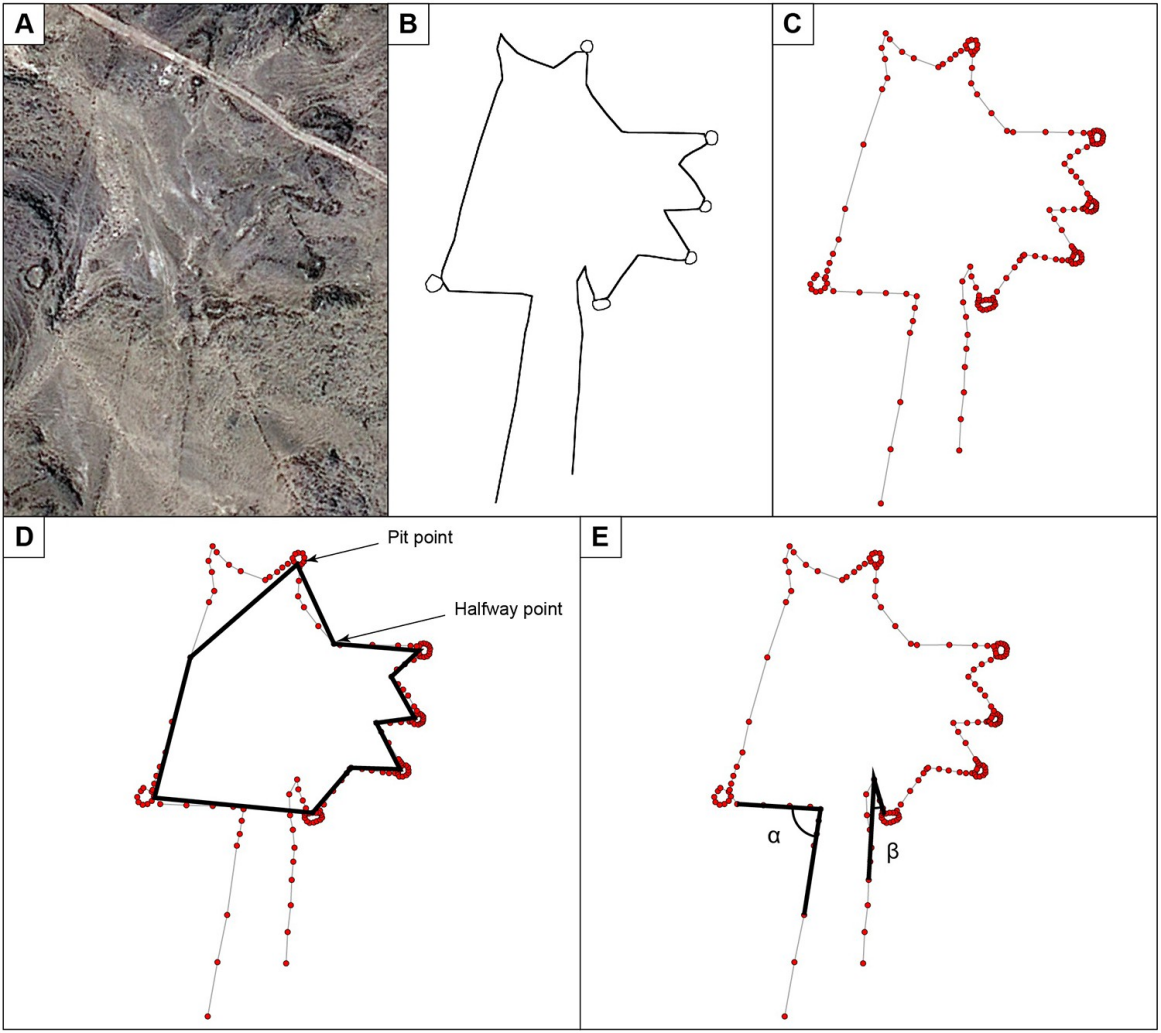
Computer-based verification using geometric graphs. (A) Desert kite satellite photo. (B) The kite extracted from the satellite photo. (C) The geometric graph extracted from the kite. (D) Polygon representation of the enclosure shape. (E) External angles between the driving lines and the enclosure.

In order to quantify the degree of similarity between the kite engravings and the archaeological kite structures, we undertook a computer-based verification. We compared each engraving with 69 archaeological kites from various areas (S5 and S6 Tables). To do so, we used a graph modelling of the kite structures from satellite images (Fig 10A–10C). Graphs, which are mathematical structures composed of sets of vertices linked by sets of edges [20] are commonly used to model objects and shapes in several domains, including kite studies [21, 22].

We represented the lines by edges and the ending points of the lines by vertices. A kite graph is an attributed undirected weighted graph *G* = (*V*, *E*), where:

- Each vertex *v* ∈ *V* has two attributes *x* and *y* which represent the coordinates of the vertex in the Euclidean space. Note that *x* and *y* also represent the coordinates of the corresponding pixel in the satellite image.
- Each edge *e* = (*u*, *v*) ∈ *E* has a weight which corresponds to its length. Let (*u*_*x*_, *u*_*y*_) and (*v*_*x*_, *v*_*y*_) be the coordinates of vertices *u* and *v* respectively, the length *ω*_*e*_ of edge *e* = (*u*, *v*) is computed as follows:

$${}_{e}=\sqrt{{\left(u_{x}-v_{x}\right)}^{2}+{\left(u_{y}-v_{y}\right)}^{2}}$$ (1)

With this modelling, comparing kites becomes a problem of geometric graph comparison. Several similarity measures are proposed in the literature to compare geometric graphs [23], but their efficiency is closely related to the objects they were defined to compare. Therefore, we propose a new similarity measure adapted to kites. It is based on three main kite properties considered as discriminatory by archeologists, and which are in order of importance: 1) enclosure shape, 2) the external angles between the driving lines and the enclosure, and 3) the ratio between the length of the enclosure and the length of the driving lines as expressed by this equation:

$$Sim\left(K_{1},K_{2}\right)=w_{1}*E\left(K_{1},K_{2}\right)+w_{2}*A\left(K_{1},K_{2}\right)+w_{3}*P\left(K_{1},K_{2}\right)$$ (2)

In this equation, *E*(*K*_1_, *K*_2_) measures the similarity between two enclosures, *A*(*K*_1_, *K*_2_) compares the angles between the driving lines and the enclosure and *P*(*K*_1_, *K*_2_) compares the driving line and enclosure lengths for the two compared kites *K*_*1*_ and *K*_*2*_. *w*_1_, *w*_2_ and *w*_3_ represent the importance given to each criterion. They are numbers between 0 and 1 and their sum is equal to one.

The enclosure is the most important part of the kite structure. The main properties distinguishing two enclosures are the number of pit-traps around the enclosure and the enclosure shape. We capture the shape of the enclosure by a polygon defined by the vertices corresponding to the pit-traps and vertices half-way between two pit-traps (Fig 10D). To compare two enclosures, we focus on the shape of the corresponding polygons without emphasizing the exact dimensions. Then we represent each polygon by a string of angles and directions and we use string edit distance [23] to compare them. The angles between driving lines and the enclosure are also important properties of the kite structure (Fig 10E). To compare these angles for two kites, we use simply the difference between their values within (*A*(*K*_1_, *K*_2_). Finally, to take into account the lengths of the enclosure and the driving lines into our similarity measure, we consider, within (*P*(*K*_1_, *K*_2_, the difference between their ratios, i.e. the ratio between the length of the enclosure and the length of the driving lines.

S5 and S6 Tables present the similarity scores we obtained when comparing the engravings with the archaeological kites, using this similarity measure. The scores are given as percentage of similarity and the obtained values range from 26.83% to 81.43% for the Jebel az-Zilliyat engraving and from 11.46% to 75.90% for the Jibal al-Khashabiyeh engraving. The higher the score, the higher is the similarity between the kite and the engraving. According to this, the most similar kite to the engraving found in Jebel az-Zilliyat is the kite AB135, located 2.30 km from the site where this engraving was found, confirming that the engraving is most likely a reproduction of this kite. For the engraving found in Jibal al-Khashabiyeh, the most similar kite is JKSH 01 (with a similarity score of 75.90%), located 16.3 km away from the engraving, while the second most similar kite, JKSH 07 (with a similarity score of 73.52%), is only 1.4 km away from the engraving (S5 and S6 Tables).

## Discussion

### Similarity between neighboring kites and engravings of kites and plans to scale

Comparisons between kite shape and the engraved representations of kites show clear similarities, easily observed with qualitative and quantitative approaches. The engraved designs represent neighboring kites both in Jordan and Saudi Arabia. The engravings are surprisingly realistic and accurate, and are moreover to scale, as observed by the geometric graph-based assessment of shape similarity. Kites are gigantic structures and are extremely difficult to apprehend in the field without access to a comprehensive image of their layout (an aerial view is the most appropriate). Yet these engravings are obviously mental depictions that could only have been made by their users and/or builders. At Jebel az-Zilliyat, the number of pit-traps on the eastern representation is undoubtedly similar to the original state of the AB135 kite (Fig 9B). As mentioned above, the two southern pits are both at a very specific location along the edge of the escarpment. The better-preserved of the two engravings in the vicinity also shows the two southern cupules on another plan of the boulder, giving the depiction of a realistic three-dimensional aspect. The orientation of the engravings on an unmovable boulder is also the same as that of the nearby kites, and finally, the engraving depicts a pair of kites, and two pairs of kites are the closest structures to it. The kites are depicted at a scale of approximately 1:175 and are in keeping with cardinal directions. At Jibal al-Khashabiyeh, the engravings are also similar to the kites, with a similar number of pit-traps to kite JKSH 01 (S7 Fig). Overall, the shape, layout and proportions of the kite engraving are consistent with the actual remains of nearby kites (Fig 9A). The schematized shape of the drawing fits surprisingly well with the top-view drawings of the structures. In particular, the proportions between the corridor width delimited by the driving lines and the diameter of the enclosure are similar to those of kites JKSH 01 and 04. Based on this comparison, the kite carving at JKSH was designed at a scale of approximately 1:425. The pit-traps in turn are significantly oversized on the drawing compared to their actual size (approximately from two to five times bigger). However, this is not surprising as these features would have been barely perceptible at the drawing scale, and certainly too small to be accurately carved in the stone. Moreover, our study and analysis showed that these features form the final trap and are thus the focal point of the hunting device where animals would accumulate [7]. The exaggeration of pit-trap size in the kite depictions could therefore also be interpreted through the prism of symbolism, in view of the significance of these features.

### Other maps, plans and representations of mega-structures in human history

Up until now, no cases of accurately depicted Neolithic architectural structures by those who designed them were known in the archaeological record. These examples of kite representations are thus the oldest known architectural plans to scale in human history. The only older or contemporaneous examples of mental representations of space are schematic maps of the natural surroundings of human groups throughout recent prehistory. Back in earlier periods, some Upper Paleolithic engravings from Europe have been interpreted as maps, used for portraying hunting strategies in a favorable topographic context of geographic bottlenecks and natural barriers, for example at Abauntz Cave in Spain, at Kiev-Kirillovskaya in Ukraine, or at Pavlov and Předmostí in the Czech Republic [24–29]. These are certainly examples of symbolic mental representation, but they are abstract representations, not scaled depictions of a landscape. Recent work interpreted a Paleolithic depiction at Molí del Salt campsite in Spain [5] as a naturalistic depiction of a reality directly facing the artist. This cannot thus be considered as an actual map/plan, although it is convincing enough to be construed as a mental representation of a social space. Some of the earliest documents described in Europe as actual map representations are the agricultural plot depictions from the Bronze and Iron Ages known from the Alps, at Mont Bégo or Val Camonica notably [30–33]. These are complex representations of agricultural landscapes including various field features, enclosures, and dwelling areas, as well as road networks. However, it is unclear as to whether they represent real landscapes in the vicinity of the engravings, or more distant, or even imaginary landscapes. In another region with rich or complex representations of probable maps, a probable slightly older depiction of a whole area was found on a massive schist slab from Saint-Bélec in western France. The slab bears what appears to be a real cartography of a territory, with natural details such as mountains and rivers, and anthropogenic structures such as burial mounds, enclosures, or settlements [34].

The advent of the Neolithic at the beginning of the Holocene in the Near East saw the progressive development of sophistication and specialization in built dwelling and utilitarian mega-structures and megalithic constructions, in parallel with major social and economic changes such as domestication, sedentarism, agriculture, societal hierarchism and the development of long-distance trade (e.g.: [35, 36]). Nevertheless, only a few depictions of social spaces and models of large-scale objects are known. A mural from level VII of Çatalhöyük (Turkey), dated to approximately 6,600 BC, is another example of a possible map, similar to the Upper Paleolithic specimens. It depicts a village with a possible reference to a volcanic eruption of Mount Hasan Dağı [4, 37–39]. The oldest three-dimensional model of a large-scale object is a sea-faring reed-bundle boat, known from the Ubaid-period H3 site in Kuwait, dated to around 5,000 BC [2]. Furthermore, the oldest models of built dwelling structures made of burnt clay are known from the Kodjadermen-Gumelnița-Karanovo culture in southeast Europe around 4,500 BC [3]. All are fascinating examples of mental representations, in two or three dimensions, but are not comparable to the kite plans to scale. Comparable plans, at least intended to be to scale, can only be found much later during the third and second millennia BC in Mesopotamia, with for example the inscribed tablet of Yorgan Tepe dated to 2,300 BC [40], or the one from Nippur dated to 1,500 BC [41]. They both represent almost cadastral maps, sometimes with rivers and mountains. Once again, they do not reach the degree of accuracy of the kite engravings.

Other not-to-scale depictions of kites are however known elsewhere in Jordan [42–47], Syria [48] and Uzbekistan [49], from undated contexts. Nevertheless, they are schematic and do not reveal such realistic details as the Jibal al-Khashabiyeh and Jebel az-Zilliyat examples. For instance, none of them depicts the pit-traps as hollow carved three-dimensional shapes. Instead, pit-traps are rather simply represented by circular two-dimensional shapes. But other crucial information is sometimes present on the not-to-scale examples, such as depictions of human and animal figures inside and outside the kite structure. Jordanian (Cairn of Hani and Wadi Hashad) and Syrian (Hemma) engraved rocks show herds of small horned mammals, in all likelihood gazelles, wild goats or ibexes. Only a few such engravings of animals have been found in the vicinity of Jibal al-Khashabiyeh, and much more abundantly around Jebel az-Zilliyat, but they were never directly associated with specific kite representations. In S12 Fig, a series of previously known kite engravings is presented. In the top row (A and B), the two engravings show hunting scenes where animals and hunters are exaggeratedly depicted, they are also simplified and the kite is figuratively represented (especially in A, where for example the driving lines are symbolized by hatched lines). In the middle row (C, D, and E), only the distal part of the whole kite is represented, with almost no dedicated space for the driving lines. The pit-traps are very clearly exaggerated, probably to signify their functional importance. In E, the pit-traps are contiguous all around the periphery of the enclosure, which is never observed in desert kites, at least in the region where the engraving was found. Finally, in all cases, it was not possible to clearly associate one kite in particular that could have been represented by one engraving, contrary to what we found in the present study with the plans to scale of well-identified neighboring mega-structures.

### Why represent kites to scale? Construction planning, collective hunt organization or symbolic aspect?

The construction of kites always has to deal with the generally complex local topography, and the different functional elements require careful arrangement by playing on convexities and breaks in slope [7, 9, 12, 14, 16, 49]. The trap incorporates the topography in which it is designed; it is both a complex object and a spatial object. The dual nature of this feature suggests the necessity for spatial information to explain the engraving of a plan. Two different functional uses of this plan can be investigated, in the frame of genuine hunting architecture [50]: a layout plan to plan the construction of the kite, or a descriptive plan to organize the collective hunt. Thirdly, a symbolic dimension may also come into play, as with other known representations of kites.

In the case of a kite construction plan, detailed knowledge of the terrain and animal movement in the complex topography would have led to the formalization of the trap and its graphic representation before the construction phase. This would have ensured that the structure was in accordance with the project during construction. However, in the cases studied here, the surface of the engraved rocks does not show, in reduction, the topographical details on which the construction was clearly based. It therefore seems unlikely that the physical materialization of the trap would have endeavored to follow the engraved plan, especially as determining topographic elements are not depicted. However, if this were the case, it would imply that these elements had been identified beforehand, but the fact that they are absent from the plan contradicts its very purpose. This is different from a modern development plan in which the topography is surveyed beforehand and then plotted.

The second hypothesis of a plan for preparing hunting activities is more plausible. In such a case, the graphic representation of the various functional elements making up the trap would formalize the process, to determine, for example, the number and position of hunters, to coordinate their actions and to anticipate animal reactions. A map would most probably be used here as a means of communication (almost like an ancestral way of writing) and would enable the collective interaction required for the smooth running of hunting operations: designing hunting/trapping strategies, understanding and explaining the architectural and topographical specificities of a structure that cannot be seen from the ground as a whole, and is only known to the builders and designers of the mega-trap. The use of the edge of the engraved rock to signify that certain pits are hidden by the break in the slope (at Jebel az-Zilliyat, Fig 8B), or the chevron drawings (at Jibal al-Khashabiyeh, Fig 5C), are simplified but useful representations of the topography. Here, we perceive the principle of modern cartographic symbols: spatial features (here topographic details) are not drawn by scaling their shape but located and represented by symbols. However, the meaning of these symbols is difficult to interpret, such as the zigzag pattern at Jibal al-Khashabiyeh and the locked-point pit-trap type at Jebel az-Zilliyat. These details may have been useful in explaining or planning the functioning of the trapping devices, but no clear-cut explanation can be advanced, as of yet. The depiction of two pit-traps along the cliff at Jebel az-Zilliyat also indicates the use of the natural topography in hunting strategies and kite constructions. The engraving of these features is clearly significant as the three-dimensional rendering on the kite plan indicates a specific purpose in the whole mega-trapping system. As a matter of fact, slope breaks and cliffs were frequently used to build pit-traps, almost everywhere in the kite global distribution zone. Such natural features served for using the different topographic levels to circumvent or reduce the effort of digging the pits too deep, and for dissimulating the presence of the pits [7].

This being said, a simpler diagram, where the elements are not necessarily to scale, but characterize and represent the different areas, could also be more effective than a scaled plan in the case of a purely practical use of this graphic representation. Thus, these engravings could also be interpreted as the consequence of a quest for a very advanced and sophisticated mastery of the knowledge of perceived space, which would be directly linked to the command of lived-in space. The latter ends up being overcontrolled by the construction of increasingly larger and more effective structures for trapping large numbers of wild animals. In short, these representations also bear symbolic significance, like an almost patrimonial need to demonstrate knowledge, to pass on knowledge of space (kites are built on and around precise topographical criteria, for example), of what this space contains (the ability to hunt masses of animals as a result of excellent knowledge of animal behavior and migration seasons), and of how this knowledge can be harnessed (building mega-traps).

However, these engravings should perhaps not be solely construed as symbolic depictions. A utilitarian function remains a credible hypothesis, either for planning kite construction or organizing collective hunting. These utilitarian and symbolic explanations for engraving kites to scale are not incompatible and could well be interconnected.

### Questioning the evolution of the perception of space and the conceptualization of gigantic shapes

Three sometimes simultaneous driving forces have presided over the development of cartography throughout its history: the emergence of a need, the invention of a technique, and the emergence of a worldview.

Firstly, the emergence of a need is a driving factor in the development of cartography. Such needs may arise in the case of territorial defense or tax collection, for example [51, 52]. In the case of kite engravings, as we have just seen, the need exists and could be sufficient motivation to develop a plan. This need is not necessarily exclusively practical and may be driven by the strong symbolic dimension intrinsic to kite structures. With the emergence of highly specialized hunting techniques, generating innovative and unprecedented strategies for acquiring animal resources, it is likely that kites were a significant cultural feature of these hunting societies, and that their economy and lifestyle were largely determined by these structures. This socio-cultural or even identity-building dimension may have stimulated a symbolic need for a technically advanced representation of these remarkable structures, which were probably also territorial symbols.

Secondly, the invention of a technique is also a driving force in the development of cartography, for example the invention of the compass which gave rise to portolans in the sixteenth century, or the invention of remote positioning in contemporary geomatic applications [53, 54]. The question of the techniques used to produce the engravings arises, as does that of the construction of kites at a scale far exceeding any architectural undertaking ever carried out by a human group. Fieldwork by present-day archaeologists shows that the morphology of a kite cannot be understood without recourse to aerial, satellite or planimetric support on account of its size and topographical complexity. Of the hundreds of kites studied in the field, it has never been possible to take a photo from the ground showing the entire enclosure, or the rest of the structure, such as the layout of the driving lines as a whole. A walker whose route would encounter a kite would not suspect the presence of a single, coherent layout. And without tools (tacheometer or GPS), a topographer would be hard pressed to produce a sketch of a kite as geometrically reliable as the engravings. It would therefore seem that kite-building hunters knew how to use a surveying technique, still unknown to us, involving notions of measurement and even calculation. The technical challenge of building a kite can only echo the appearance of these planimetric scaled representations. These two innovations—the construction of the largest structures ever made by human at the time, and cartographic representation to scale—are thus closely interdependent. The common denominator of these innovations is the need to master the three-dimensional perception of a territory or space (landscape). This prerequisite is essential both for the planning of constructions as vast as kites in a complex topographical environment, intended for the capture of animals whose behavior and movement patterns in the landscape must be known, and for the practice of hunting with these traps. We postulate that this major advance was also at the root of significant developments in the planimetric representation of space and landscapes, leading to the production of the first plans to scale.

Finally, the emergence of a worldview is a third vector of cartographic evolution, like the map of Eratosthenes or Ptolemy’s Geographia [55]. In the case of the kite engravings, there is no need for a worldview in terms of scale. The aim is not to represent a vast territory, let alone the known world, but only the area of the layout. However, this requires decentering, a view from the sky, revealing an elaborate abstraction of space. The process of reducing a space, the elaboration of analogies between physical space and graphic representation is an important development in abstract thought and symbolic representation [1]. In relation to the Çatalhöyük representations, which are somewhat more recent than the kite engravings, Meece [56] questions the ‘map’ status attributed to them. Meece assumes that the cognitive capacities required for such an imaginary representation are too great, requiring a shift from a view of individual points of view to a view from above, following the assumption of Gartner [57] that maps can only be made under conditions where aspects such as agriculture, private property, long-distance exchange, conflict, tribal relations and other aspects of redistributive economies are present. Indeed, work describing early formal maps (in Mesopotamia [41], the central Andes [57], China [58]) concerned complex literate societies with a tradition of written records or with trade relations with other literate societies. These documented examples of formal cartography are relatively late, and no cartographic representations were previously known in the prehistory of the Near East or even in any Neolithic culture. It was also thought that small, landlocked societies had little need for spatial information [59] and therefore did not need maps for pathfinding or information storage.

However, in the same way as cases of empirical knowledge from Mesopotamia or the central Andes, a disjunction of human perception from ‘being in the world’ to ‘being above (or even beyond) the world’ [60] clearly underlies the kite engravings. This tends to call into question the idea that a complex literate society is a necessary condition for map-making. Indeed, we can consider that a need for complex spatial information, rather than literacy and a tradition of written production, is crucial for cartographic production. This need arose in societies that had to resolve complex management issues themselves, such as the social organization of agricultural land or urban planning. But it also occurred in the case of simpler, small-scale societies, in order to resolve other spatially and socially complex issues. There are many examples throughout the world of mental representations of space made explicit by their creators to outside observers ignorant of the spatial meanings of what may appear to be abstract artistic works (e.g.: Aboriginal lines and spirals to represent paths connecting water points [61–63], Tuareg cartographic representations of the relative positions of several towns in an area of more than 2,000 km [64], or those of the Snake River by Native Americans representing a 600-km-long area [65]). This situation in traditional pre-literate societies does not contradict the fact that the emergence of this type of representation can only occur within specific social structures [60]. Devising a collective hunting strategy would thus present these characteristics of complexity, with the need to spatially formalize hunters’ actions on the one hand, and to communicate collectively around this formalization on the other.

## Conclusions

The discovery of two prehistoric engravings, one in the northern reaches of Nefud in March 2015, the other in south-eastern Jordan in June 2015, provides some answers to these complex questions of the representation and conception of space, faced by human groups since ancient times. These examples of representations both show, in a similar style, images of kites that can be described as planimetric reproductions. They were found in the vicinity of studied and excavated kites dated to the Neolithic period. These two engravings are thus the oldest planimetric representations to scale known to date. Moreover, desert kites appear to be the earliest stone-built constructions known up until now at such a large scale. On account of the size, organization, functionality, and effort required for their construction, these mega-structures should actually be considered as fixed infrastructures of the landscape. We believe that the technical challenges involved in the construction, use and maintenance of kites, and their strong sociocultural value in the context of Neolithic developments are at the essence of the earliest planimetric representations known to date. These engravings also teach us that the technical progress required for the construction of kites (large-scale construction requiring a developed perception of space and inscription in the topography), is at the origin of progress in techniques of planimetric representation, as a logical continuation of skills acquired in the perception of space in general. The community aspect of these large-scale hunts is another element supporting the hypothesis of a need to communicate, in particular to share spatial information, by means of a realistic representation intended for a human group participating in a common action.

The precise history of kites is not yet known across the whole, very vast, kite distribution area. It is, however, now clear that with regard to knowledge of human/animal relationships—and more generally the evolution of subsistence modes in a region of the world where sedentarism and the urban phenomenon emerged—the significance of these structures has been underestimated until now. The discovery of these very ancient representations highlights the question of the methods used by kite builders. As far as construction methods are concerned, the walls were built with care and ingenious use was made of topographic features, but the techniques used are still simple and basically revolve around the assembly of unsquared stones of various sizes. In contrast, the layout of the entire system was based on relatively elaborate methods, hitherto unknown for the periods under consideration. Whereas kites could be built by marking out the visual range of a place, the crux of the matter here is the very design of the kite in its natural environment. Kites are large material structures that could not be designed without what we call today planning. Yet this is an intellectual construction, an abstraction calling upon an elaborate representation of space, which was very difficult to grasp up until now, due to the paucity of data on kites. The visual representation of lived-in space, perceived space, dreamed space, has existed for a long time, as far back as the first ‘artistic’ representations (naming them in this way avoids giving them a utilitarian or clearly symbolic role) from the Middle Paleolithic or Middle Stone Age in Africa, and more figuratively from the beginning of the Upper Paleolithic in Europe or Australia. But the notion of an aerial view, now widely used since the western perception of the bird’s eye view of a territory, forms the basis of western cartography, and represents our exclusive conception of the territories that surround us. This notion did not make sense in traditional pre-literate societies that represented spatial information symbolically.

These two major innovations, i.e., building what would become the largest structures in human history at that time and making cartographic representations to scale, are closely linked by a common point: mastering the three-dimensional perception of a space, and translating it into an inscribed form of communication. This continues to challenge our modern perception of the lived-in, perceived, and dreamed space, versus the represented one. The discovery of these examples of early accurate cartographic representations has far-reaching consequences for our understanding of the evolution of human cognition. The transposition of a mental conception of (a large, not possible to grasp as a whole) space onto a two-dimensional (small) surface inherent in these representations, is a milestone in intelligent behavior, progressively acquired from the early stages of human evolution.

## Materials and methods

### Site identification, survey and excavation

At Jibal al-Khashabiyeh, kites were discovered in 2013 and recorded and excavated in 2015 and 2016 as part of the South-Eastern Badia Archaeological Project (SEBAP), in cooperation with the Department of Antiquities (DoA) of the Jordanian Ministry of Tourism and Antiquities. They were recorded with two systems: the one used in the field by SEBAP (JKSH 01 to 08) and the one used on global satellite imagery interpretation by the Globalkites Project (JD1088 to 1095; for Globalkites methodology see [6, 7, 13]). At Jebel az-Zilliyat, kites were discovered in 2014 and excavated in 2015 as part of joint fieldwork by the Dumat al-Jandal archaeological project and the Saudi Heritage Commission. Kites were recorded in two systems: the Dumat al-Jandal database (DAJ137, DAJ138, DAJ139, DAJ140) and the one used by the Globalkites Project (respectively: AB135, AB136, AB547, AB549). In Jordan, excavation and survey permits for the 2015 and 2016 seasons were issued by the DoA (Num. 2015/31, 2016/48). In Saudi Arabia, the Saudi Heritage Commission issued the excavation and survey permits for the 2014 and 2015 seasons.

The locations of each archaeological structure and feature were recorded in the field using high-precision Trimble Pathfinder Pro XRS Differential Global Positioning System (DGPS). Geomatic analyses of both fieldwork data and satellite imagery went through a Geographic Information System (GIS; using Esri ArcGIS software). A regularly updated online interactive map shows the current number of recorded kites (available at www.globalkites.fr). DGPS mapping and kite aerial photography (KAP; using HD digital cameras triggered by intervalometer or radio control system) led to Digital Surface Models (DSM) and photogrammetric rendering of topography and archaeology (with Agisoft PhotoScan software), documenting archaeological features visible on the surface, and archaeological excavations.

Excavations were carried out by hand, usually in a half or a quarter of the pits surrounding the kite enclosure. All sediments were systematically dry-sieved (0.5 cm mesh; 80 to 100% of the total volume) to retrieve archaeological material, while geoarchaeological stratigraphic studies required the collection of small dust samples and large soil samples. Dust samples were analyzed under the petrographic microscope (Olympus BH2, x200 to x400) for mineral, anthropogenic and/or biogenic components. Larger soil samples were water-washed (0.5 mm mesh), observed and sorted under the stereomicroscope (x10) for biogenic material (radiocarbon datable elements).

Both engraved stones were discovered in 2015 during pedestrian surveys, by Juan Antonio Sánchez Priego and Wael Abu-Azizeh in Jordan and by Charly Poliakoff in Saudi Arabia.

### Analysis of the engravings, and photogrammetry

The analyses of the macroscopic designs and of the technical parameters were based on various works (e.g.: [66–68]), directly by looking at the engravings, and also by carefully studying photogrammetric models, whose production is described hereafter.

The Jibal al-Khashabiyeh engraved stone was handled post fieldwork, as the stone was mobile and removed as important artefactual evidence. It was therefore processed for photogrammetry in a controlled-light environment, using spotlights with appropriate orientation. The stone was photographed from the front (with the engraving) and back (opposite of the engraving) from multiple view angles (total 132 photos). Ten markers (materialized by small white paper tapes with a dot mark inside) were positioned on the lateral sides of the stone, at regular intervals all around the stone (three markers on each long side, two markers on each of narrower top and bottom sides), allowing subsequent alignment and merging of the distinct 3D model chunks of the two sides of the stone. The 3D model was processed using PhotoScan (Metashape) Agisoft software v.1.6.1. (with the following settings: Align accuracy: High; Dense cloud quality generation: High; Mesh build: custom 4 000 000 faces; Texture size/count: 12288 x 1). In view of the quality of the photos, the 3D model underwent minimal standard cleaning processing during the various building steps. The model was set to scale through measurements taken on the marker points set on the stone prior to photography.

The 3D model was then set into a defined orthonormal frame, with its origin set at the center of the stone, the x axis through the width of the stone, y axis through its length and z axis through its thickness. Orthomosaics were generated for six predefined views (front, back, left, right, bottom and top), producing a basic plate with all faces of the stone (Fig 4C). This document clearly illustrates the location of the drawing according to the relief of the stone, especially on its curved long edges.

We undertook additional processing steps to produce a projected view of the engraving. The engraving is made on a roughly parallelepipedal monolith, which was worked (carved) on the edges producing irregular curved sides. Important parts of the engraving (the corridor-like shape of the kite driving lines, as well as three of the cup marks materializing the pit-traps) were voluntarily drawn using the curved edges of the stone to emphasize their topographical situation. The production of a projected view of the engraving in its entirety required therefore a specific technical approach. A total number of 260 successive orthomosaics of the stone surface were produced, starting from the front predefined view, and rotating the stone only a few degrees all around (using the x axis of the trackball in order to maintain a consistent y axis). The 260 views produced over a rotation of 360° were made at a rotation angle of less than 1.5°. All these orthomosaics were then processed with Adobe Photoshop. Only a central 3-cm-vertical strip was kept for each one (as the central part of the image is less subject to projection distortions), and they were manually stitched together using the overlapping edges of each strip. This process generated a fully deployed projected view of the stone, revealing the whole kite engraving plan (S5 Fig). This document was used as a basis to draw the full layout of the kite engraving on a two-dimensional plan (Fig 4D), which in turn allowed for comparisons of the carved stone kite representation to actual top-views of the nearby kite structures (Fig 9A). Additionally, a 3D model was produced, and is manipulable at: https://doi.org/10.34969/cnd3d/737849.o.2022. The stone was weighed on a scale (Health-o-meter Professional): its weight is 92 kg, which roughly corresponds to an estimation based on an average limestone density of 2.6 g/cm^3^ and a volumetric measurement of the monolith on the 3D model of 36,768 cm^3^.

The engraved boulder at Jebel az-Zilliyat has not been removed from its original location. Several photosets (total 170 photos) have been made in the field under different lights and angles, with one 50 cm photo scale for geometric point of reference. Two photosets were dedicated to the engraved details, and one to the general context; all have been merged into the general 3D model of the entire setting. The 3D model was processed using PhotoScan (Metashape) Agisoft software v.1.6.1. using two chunks, one for the details and one for the modeling of the whole block. The first chunk incorporated high-resolution alignments of all photosets. It was first exported without points of reference, as a digital terrain model (DTM) and an orthophoto, planar view. These two documents were put to scale with ArcGIS into a three-dimensional Cartesian coordinate system, with axis lines x, y and z. Georeferencing information are then accurate in the sub-millimeter range. With PhotoScan, three points of reference were then transferred into the first chunk, in order to obtain a dense cloud and a high-resolution model to scale. From the dense cloud, a DTM and two orthomosaics were generated, one with the photos taken in sunlight, another without any cast shadows. From the DTM, in ArcGIS, we produced a shaded image, showing black and white visualizations with low-angled light. It was made through four lighting angles (45°, 135°, 225°, 315°), revealing all engraved designs, even the most tenuous ones (S9 and S10 Figs).

For producing a projected view of the engraving, procedure was the same as the one of Jibal al-Khashabiyeh. As the boulder surface at Jebel az-Zilliyat was in this case almost flat, except for the two representations of pit-traps in the southeastern part of the rock, only 13 exports as DTM and orthophotos were generated in planar mode, with a rotation less than 1.5° on the x or y axis of the trackball. Obtained files were turned into shaded mosaics with Adobe Photoshop, obtaining the most legible version of the engraving.

To model the whole engraved stone into the same coordinate system, 16 points of reference were transferred into the second chunk with PhotoScan. In this local system, a dense cloud was generated and then cut according to the first chunk’s limits, to merge both dense clouds, and to incorporate to the contextual ensemble the high-resolution one of the engraving. This merged dense cloud is made of 157,655,331 points. A first 3D model was made from this cloud, including 30,461,629 faces. To produce a PDF file out of this 3D model, it was reduced to 182,321 faces. The model texture was generated from sunlight-exposed photoset as it offers more contrast for a lighter file (manipulable at: https://doi.org/10.34969/cnd3d/490124.o.2022.

### Petrographic analysis for Jebel az-Zilliyat boulder

At Jebel az-Zilliyat the petrographic characterization of the block is based on the observation of fresh breaks with a stereomicroscope and on the mineralogical analysis of the grains with a petrographic Olympus BH2 microscope (see Supporting information).

### Radiocarbon dating and optically stimulated luminescence dating

At Jibal al-Khashabiyeh, charcoal remains were recovered from the deep stratigraphic fill of the kite pit-traps. They were either charcoal from small hearths, indicating a later reuse of the structures as ephemeral shelters, or fragments of residual charcoal trapped in aeolian sediments. They were sampled at different depths of the sedimentary fill. Seven samples were processed for AMS radiocarbon dating at the AMS Laboratory of the University of Arizona. The chronometric dating results (S3 Table) are presented in detail elsewhere [8]. At Jebel az-Zilliyat, *Arnebia* sp. calcitic nutlets (7 to 11 small fragments per sample) were used for radiocarbon dating. They were sampled near the bottom of each stratigraphy. The two samples were processed at the Poznań Radiocarbon Laboratory (Adam Mickiewicz University). All dates were calibrated with Chronomodel v.2 [69] using IntCal20 atmospheric calibration curve [70].

OSL dating was carried out on sediment samples taken from the filling of trap pits. These ages will post-date the age of construction and final use. Small aliquots of quartz (1 mm) were employed for determination of Equivalent Dose (D_e_) using a modified Single Aliquot Regenerative Dose (SAR) protocol [71]. Mean D_e_ values were calculated using the Minimum Age Model [72] due to the presence of partial bleaching as observed in the D_e_ distributions. The concentration of dose rate relevant elements was determined using high-resolution gamma spectrometry (cf. [73]), annual dose rates and ages were calculated using ADELE v.2017 software [74] (see S11 Fig and S4 Table).

### Graph modeling studies

We did not use an automated image segmentation algorithm to compute accurate similarities between the kites and the engravings. Kite shapes had to be extracted manually to deal with the numerous reliefs present in satellite images of kites and obtain accurate kite representations. Thus, kite shapes were manually extracted from images by placing points all along the kite and linking them by drawing a line between two consecutive points (Fig 10A and 10B). The remaining tasks are fully automated by our algorithms. We modeled each extracted shape on a graph where edges are the lines, and vertices are the ending points of lines (Fig 10C). Each edge is weighted by its geometric length. To compute a similarity score between two kites, we also used an algorithm that extracts the shape of the enclosure and the external angles between the driving lines and the enclosure (Fig 10D and 10E). All the algorithms are implemented using Python 3.6. The source code of our algorithms as well as the data (vectorial images of the kites and their corresponding graph representations) are available on https://gitlab.liris.cnrs.fr/aekiouche/kite-project. S5 and S6 Tables present the similarity scores we obtained when comparing the engravings with the archaeological kites.

### Inclusivity in global research

Additional information regarding the ethical, cultural, and scientific considerations specific to inclusivity in global research is included in the Supporting Information.

## Supporting information

S1 Appendix Supplementary text. (PDF)

S1 Fig Kite JKSH 04 (JD1091). Top plan and different views of the excavation of pit trap St.02.
(JPG)

S2 Fig Kite DAJ137 (AB135). (A) Aerial view of pit-traps L03 (archaeological excavation), L04 and L05. (B) L03 before excavation. (C) L03 after excavation.
(JPG)

S3 Fig Kite DAJ140 (AB549). Aerial oblique view of the enclosure.
(TIF)

S4 Fig Kite DAJ140 (AB549). Aerial view of pit-traps L01 (archaeological excavation, on the left) and L02 (on the right).
(JPG)

S5 Fig Composite ortho-mosaic illustrating the fully deployed projection view of the engraved monolith found at JKSH F15 site. An outlined drawing shows the interpretation of the whole engraving.
(JPG)

S6 Fig Hypothetical interpretation of the chevron pattern engraved on the kite depiction on the monolith from JKSH F15 site, Jordan as a topographic symbol. The detail of the chevron pattern (bottom) is compared to the slope break seen in the topography (here at kite JKSH 04, top).
(JPG)

S7 Fig Reconstruction of the layout of the enclosures at the three excavated kites in Jibal al-Khashabiyeh, Jordan. The reconstruction is based on the structural remains preserved in the field to estimate missing pit-traps around the enclosure’s perimeter.
(JPG)

S8 Fig Comparison between the engraving and a desert kite from Jebel az-Zilliyat, Saudi Arabia. (A) Top-view and profiles of the southern part of desert kite AB135. (B) Top-view and profiles of the southern part of the engraving.
(JPG)

S9 Fig Various angles of light to show details of the engraved eastern kite from Jebel az-Zilliyat, Saudi Arabia. From the DTM, in ArcGIS, we produced a shaded image, showing black and white visualizations with low-angled light. It was made through four lighting angles (45°, 135°, 225°, 315°), revealing all engraved designs, even the most tenuous ones.
(JPG)

S10 Fig Various angles of light to show details of the engraved western kite from Jebel az-Zilliyat, Saudi Arabia. From the DTM, in ArcGIS, we produced a shaded image, showing black and white visualizations with low-angled light. It was made through four lighting angles (45°, 135°, 225°, 315°), revealing all engraved designs, even the most tenuous ones.
(JPG)

S11 Fig Characterization of OSL signal properties and individual De distributions. (A) Natural and laboratory irradiated OSL signals show almost identical decay shape with a dominance of the fast component (exemplified for AJR1). Inset: De values are in the close-to-linear range of signal growth. (B), (C), and (D) De distributions plots with density probability functions for samples AJR1-3 reveal a distinct peak in De values but some values at the higher edge that are interpreted to represent incomplete resetting of the OSL signal in quartz grains of some of the aliquots.
(JPG)

S12 Fig Examples of previously known kite engravings, that are not represented to scale and less realistic. (A) From cairn of Hani’ site, Syria; redrawn from [42]:fig. 8. (B) From Khishâm-2 site, rock В 37, Syria; redrawn from [48]: fig. 3. (C) From Wisad Pools, Jordan; redrawn from [46]: fig. 44. (D) From Azraq Basin, Jordan; redrawn from [43]: fig. 3(6). (E) From Wisad Pools, Jordan; redrawn from [47]: fig. 8. (F) and (G) are the kite engravings described in the present study, respectively from Jibal al-Khashabiyeh and Jebel az-Zilliyat, represented in this figure to be directly compared with the previously known ones.
(JPG)

S1 Table Corresponding kite numbers between the scientific teams and GPS location of the Jibal al-Khashabiyeh desert kites. (PDF)

S2 Table Corresponding kite numbers between the scientific teams and GPS location of the Jebel az-Zilliyat desert kites. (PDF)

S3 Table Radiocarbon analysis results from Jibal al-Khashabiyeh, Jordan, and Jebel az-Zilliyat, Saudi Arabia. Dates were calibrated with Chronomodel v.2 [69] using IntCal20 atmospheric calibration curve [70]. Md, Q1 and Q3 refers to the median, first and third quartile of the distribution of calendar ages. Bold characters underline the earliest date obtained for each kite (i.e. the radiocarbon terminus ante quem of the construction). All calendar dates are rounded to the nearest ten. * Unreliable sample (0.24 mg of carbon only, very large standard error): not considered.
(PDF)

S4 Table Summarized OSL dating data from Jebel az-Zilliyat, Saudi Arabia. AB135 stands for DAJ137 kite, and AB549 stands for DAJ140 kite.
(PDF)

S5 Table Similarity scores obtained when comparing kites to the engraving found at Jebel az-Zilliyat, Saudi Arabia. (PDF)

S6 Table Similarity scores obtained when comparing kites to the engraving found at Jibal al-Khashabiyeh, Jordan. (PDF)
