# Supplementary material for: The oldest plans to scale of humanmade mega-structures
Source: PLoS One. 2023 May 17;18(5):e0277927. doi: 10.1371/journal.pone.0277927 (PMC10191280; doi:10.1371/journal.pone.0277927)
Supplement: S1 Appendix — (PDF) [file pone.0277927.s001.pdf]

Supporting information

The oldest plans to scale of humanmade mega-structures

Rémy Crassard, Wael Abu-Azizeh, Olivier Barge, Jacques Élie Brochier, Frank Preusser, Hamida Seba, Abd Errahmane Kiouche, Emmanuelle Régagnon, Juan Antonio Sánchez Priego, Thamer Almalki, Mohammad Tarawneh

S1 Appendix: Supplementary text

1. Archaeological excavations

1.1. Excavations in southeastern Jordan: Jibal al-Khashabiyeh

Research in the Jibal al-Khashabiyeh area identified a chain of eight kites extending over 17 km in a northsouth alignment. The locations of the eight kites discussed in this paper are mentioned in Table S1. They are all marked by two stone alignments converging towards the escarpment. In this location, severe erosion has mostly destroyed the structural remains. However, in three cases, it was possible to identify traces of polygonal star-shaped enclosures, characteristic of kite structures (JKSH 01, JKSH 04 and JKSH 07). In June 2015, the circular structures located on the perimeter of kite enclosures JKSH 01 and JKSH 04 were excavated, in order to compare the results with those obtained on an isolated kite, discovered and studied in another study area (Jibal al-Ghadiwiyat region) [12]. There, the excavation had indeed demonstrated that the circular structure located on the perimeter of the kite enclosure was a deep pit dug into the substratum with stone-built inner walls. These results demonstrated the hunting function of the kite, and indicated that the circular structure was a pit-trap designed to trap hunted animals in a deep pit. The primary objective of the excavations carried out during the 2015 campaign was therefore to determine whether the kites in the Jibal al-Khashabiyeh area followed a similar construction pattern, particularly with regard to the circular structures visible around the perimeter of kite enclosures. The question of the function of these structures was addressed through comparisons of structural and architectural organization. Furthermore, as the circular structures were also dug deep into the substratum, their infillings with large quantities of aeolian sediment could potentially contain archaeological material and (organic or sedimentary) material for dating the structures.

1.1.1. Excavation of kite JKSH 04

Kite JKSH 04 is one of three examples where the presence of an enclosure could be clearly demonstrated at the point where the driving lines converge. The presence of a narrow corridor delimited by stone walls at the approach to the western end is characteristic of kites in the Jibal al-Khashabiyeh area, suggesting a local tradition of building such structures. The area at the opening of the stone walls also takes advantage of the steep topography, where erosion has formed a natural cirque. Although the enclosure is only partially preserved, it is clearly marked by sections of low stone walls overlooking the slope to the east, and in particular by characteristic circular cells (pit-traps). Traces of the enclosure are also clearly visible on the western slope of the natural cirque, in the form of several circular pit-trap structures. It is therefore clear that the depression was originally entirely enclosed, and the gaps observed in the enclosure's layout to the north and south are due to significant gully erosion. Two circular pit-trap structures were excavated on kite JKSH 04: St.01 and St.02 (S1 Fig).

Pit-trap St.01: The structure is delimited on the surface by a diffuse and disorganized alignment of stones, and reaches 5 to 5.5 m in diameter. Excavation of the southwest quarter of the structure showed that it was cut into the substratum to a depth of 1.5 m from the present-day surface. The walls of the structure were built in stone, sometimes on an outcrop of limestone bedrock, sometimes deeper down on the virgin sediment. Several distinct levels of the fill yielded charcoal for radiocarbon dating.

Pit-trap St.02: The second circular structure is located on a point of the star-shaped enclosure. The excavation covered the entire northern half of the structure, which was dug into the natural substrate to a depth of 1.55 m from the present-day surface. The pit walls were carefully built with large stones arranged on a base of huge blocks sometimes standing side by side (S1 Fig).

1.1.2. Excavation of kite JKSH 01

The overall organization of kite JKSH 01 is very similar to that of JKSH 04. However, it seems to have combined two converging wall systems, one facing east (JKSH 01a) and the other west (JKSH 01b), with a single enclosure located in the center. To our knowledge, this configuration is unparalleled. However, it is difficult to determine whether this corresponds to the reworking of an earlier kite (with a change in orientation), or whether it may have functioned as a whole at some point. Two circular pit-trap structures were excavated on the JKSH 01 kite.

Pit-trap St.01: The first circular structure excavated on kite JKSH 01 is located along the course of the enclosure on the eastern side of the erosion gully. The survey focused on the northwest quarter of the

structure, and revealed a similar pit layout to that of the pit traps at JKSH 04. The structure is dug into the natural substratum to a depth of 1.40 m from the present surface. The walls of the structure are built in stone: in the upper parts, the masonry consists of large randomly ordered stones depending on the variable sizes of available blocks; this masonry lies on a base of very large standing stone slabs carefully placed side by side.

Pit-trap St.10: The second circular structure excavated on kite JKSH 01 is located on the western side of the enclosure, at the point of an angle formed by the course of the enclosure wall. The entire north-eastern half of the structure was excavated. The structure is dug into the natural substratum to a maximum depth of 1.80 m. The pit walls are built with large stones randomly placed in the upper part and resting on huge stone blocks at the base. This masonry is less regular but nonetheless similar to that of the other excavated circular pit traps. Numerous charcoal samples were collected at different levels of the structure's infilling: some in the upper part of the aeolian infilling, several in the level of destruction, and finally also at the base of the infilling, close to the bedrock.

1.1.3. Excavation of kite JKSH 07

Kite JKSH 07 is located north of the chain of kites identified in the Jibal al-Khashabiyeh area. It follows a general organization that is very characteristic of the kites in this area. It is composed of two main driving lines, 1.7 km long, which converge towards the edge of the escarpment. The excavation focused on the two best preserved circular structures around the perimeter of the enclosure: St.02 and St.03. They are both located to the north of the natural cirque formed by the wadi, at the top of the slope overlooking the ravine, which explains their good state of preservation. The objective was to document the construction mode of these structures in order to establish parallels with previously excavated kites.

Pit-trap St.02: Structure St.02 is sub-circular in shape, with an average diameter of 4 m. Excavation of the structure showed that it was cut into the substrate to a maximum depth of 1.8 m from the present surface. The walls of the structure consisted of stone masonry resting on the irregular surface of the limestone bedrock. The latter was represented in particular by the presence of natural formations of huge limestone spherical boulders. The base of the lining wall of the structure, which was dug deep into the substrate, rested partly on these limestone spheres and partly on the limestone bedrock.

Pit-trap St.03: Structure St.03 is sub-circular in shape, fairly regular, with an average diameter of 4 m. It is located at the western end of the arched wall adjoining St.02 to the east, at a distance of 30 m. The maximum depth of the structure is 1.6 m from the ground level. The walls of the structure were made of a very heterogeneous stone block masonry, sometimes incorporating huge parallelepipedal stone blocks,

sometimes limestone spheres, and sometimes smaller stones to level out flat surfaces for the laying of upper courses. The excavation of the pit reached the limestone bedrock. Columnar sediment samples (10 cm intervals) were taken from the stratigraphic sections of the two structures St.02 and St.03. They were used for sedimentological analysis.

1.2 Excavations in northern Saudi Arabia: Jebel az-Zilliyat

Sporadic archaeological investigation has taken place in the Al-Jawf region in northern Saudi Arabia. An archaeological operation including surveying and excavations, in cooperation with the Dumat al-Jandal archaeological project, was carried out in March 2015, to identify and sample the desert kites known in the region thanks to high-resolution satellite imagery interpretation. A total of nine kites were visited. The location of the four kites from Jebel az-Ziliyat discussed in this paper are mentioned in Table S2. The Al-Jawf region presents a very interesting combination of landscapes and archaeological sites. Human occupation in this area ranges from the Lower Paleolithic to the present, and appears to be linked to environmentally favorable conditions [75-78]. The kites explored during fieldwork in 2015 are situated in the rarefaction zone to the south of the main Harrat al-Shaam concentration of desert kites. Our preliminary results show that they are similar to the other kites from this zone. For the first time in Arabia, fieldwork and test pits in some pit infillings provided chronological data. The sedimentary facies, derived from Devonian sandstone and quartzite, were not ideal for the discovery of biogenic radiocarbon datable material, but the rapid sandy infilling of pits, soon after their last utilization, was a major asset for OSL analysis. In the Al-Jawf region, Jebel az-Zilliyat is one of the studied areas, situated west of the Jebel alHimdi and the al-Wadi graben area. Using satellite imagery remote sensing, the team identified four megastructures at the edge of a sandstone plateau, organized into two pairs (the eastern pair with kites DAJ137 [AB135] and DAJ138 [AB136]; and the western pair with kites DAJ139 [AB547] and DAJ140 [AB549]).

1.2.1. The eastern pair of kites: DAJ137 and DAJ138

Excavation of kite DAJ137 (AB135): A small test pit excavated in a first pit-trap (DAJ137-L01) revealed a very shallow deposit with little potential for sediment sampling in an indurated and carbonated sediment. A small structure, leaning against the wall of the pit-trap and interpreted as a tomb, was also excavated. This tomb-like structure was thus potentially younger than the pit-trap. Half of the width of this structure was excavated, and no artifacts were found. The construction technique consisted of facing walls in the funerary chamber with four to five courses on one side and orthostats on the other. The filling of this small structure contains particularly soft sand with collapsed stones, indicating a post-looting infilling. Several

samples of bird eggshells were collected for dating, but with limited potential. Another test pit was excavated in a second pit-trap (DAJ137-L03, S2 Fig). This structure used the natural relief for the construction of its peripheral wall. No artifact was found but several sedimentological (including for retrieving datable micro-remains) and OSL samples were taken.

Desert kite DAJ138 (AB136): The DAJ138 kite is less disturbed and damaged than its neighbor DAJ137, and was thus potentially more suitable for excavation. Unfortunately, field observations concluded that no pit-trap contained sufficient sediments and that the general topographic context was not auspicious for good conservation. Thus, no excavations were carried out, but the kite was mapped using DGPS, to ascertain its relationship with the neighboring DAJ137 kite.

1.2.2. The western pair of kites: DAJ139 and DAJ140

DAJ139 and DAJ140 are about 3 km west of the eastern pair of kites and seem to have been used together.

Desert kite DAJ139 (AB547): After attentive observations on the different sectors of DAJ139, no pit-trap was considered suitable for excavation. All were either linked to a slope end and thus exposed to potential runoff, or contained very little sediment, precluding a secure context for geoarchaeological analyses or OSL sampling. The kite was accurately mapped with DGPS mapping, showing its direct relation with the neighboring DAJ140 kite. Field observations seem to indicate that both kites functioned together simultaneously.

Desert kite DAJ140 (AB549): DAJ140 appeared more promising (S3 Fig). A number of pit-traps were filled by sediments and seemed conducive to small test excavations. Around the kite, several rounded stonemade structures were found. At least one very large tower tomb overlooks the whole area. Other large circular structures reuse stones from the kite enclosure in one place. One pit-trap was excavated at this kite (DAJ140-L01, S4 Fig) with a view to revealing the construction technique of the pit-trap wall. A type of double facing technique appears to have been used, with conjointly positioned vertical stones. This is not the typical classic double-facing technique with a small rock filling between stone courses. This construction technique consists of vertical slab alignments, sometimes with flat horizontal four-to-five course walls, with a few larger slabs positioned over the top of these walls as a corbelled construction. No artifacts were found, but a number of sediment samples were collected, as well as two OSL samples that yielded conclusive dating results. Two other small structures (DAJ140-S1 and DAJ140-S2) were also excavated, as they reused the kite enclosure walls, in order to obtain relative chronological indications. These small quadrangular (in plan) piles of stones were built directly on the sandstone bedrock and turned

out to be totally empty. They are interpreted as ‘boxes’ or ‘storage facilities’, or even as traps for small animals.

1.2.3. Presence of lithic industries on the surface

High-density lithic scatters were found in the vicinity of each pair of kites (especially near DAJ137 and DAJ140). They consist of numerous artifacts, with a high density of tools such as thin leaf-shaped bifacial foliates, knifes, end scrapers, circular scrapers, tanged and barbed arrowheads, burins on truncations and retouched blanks. Artifacts are usually very fragmented and only lightly patinated. Their preservation status and typological and technological characteristics indicate an approximate age from the end of the Neolithic period to the Chalcolithic. Their presence in the immediate vicinity of the kites is intriguing, but they are only surface discoveries, in undatable contexts, and it is not possible to establish synchrony between the stone tool makers and kite users.

2. Petrographic analysis for Jebel az-Zilliyat boulder

At Jebel az-Zilliyat, the engraved boulder is part of a chaos of huge blocks detached from the cliff that borders the wadi bed. Its petrographic nature can be described as a porous yellow (10 YR 8/6) quartz arenite sandstone poorly cemented by calcite. A few very rare centimeter-sized pebbles are present among the well sorted medium sandy grains. These petrographic characteristics make this sandstone a material on which it is easy to make deep furrows.

The surface supporting the engraving, and the engraving itself, is covered with a very fine dark grey FeMn varnish (2.5 YR 3/0) in the process of desquamation (i.e. gradually lost to weathering) as it is the case everywhere in the area. A thin discontinuous red band (2.5 YR 4/6), some millimeters to half a centimeter thick, in which the intergranular voids are filled by clay, makes the transition between the varnish and the sound rock. If the red clayey horizon is probably a relic of ancient weathering processes, the thin desert varnish of microbial origin [79,80] implies a certain antiquity of the engraving. The current climatic characteristics are no longer favorable to the development of varnishes, as shown by the observation of local historical petroglyphs, but led to its desquamation. A less arid period, more favorable to the activity of manganese-concentrating bacteria [81], than the current one seems to have succeeded the one during which the engravings were made. From a chronological point of view, it is interesting to note that in the few kites excavated, the rock varnishes are well developed, but only present on the emerging parts of the

blocks buried in the filling of the cells. The construction of the kites as well as the realization of the engravings seems thus to be included in a less arid phase than the current one.

3. OSL dating supplementary data

Samples for OSL dating were taken by forcing opaque tubes into the freshly cleaned sediment surfaces of excavated pit-traps. Additional material was taken for dosimetric measurements. The tubes were transferred to the laboratory and opened under sub-duded red-light. The outer parts of the tubes were discarded as it might have been exposed to daylight during sampling. Samples were first wet-sieved (100-150 µm) followed by removal of carbonates (20 % HCl) and organic matter (30 % H_2O_2). The quartz fraction was enriched using heavy liquids (LST FastFloat©) with densities of 2.70 g cm^{-3} and 2.58 cm^{-3} and etched in 40% HF for 60 min with subsequent >1 hour 10% HCL treatment to remove fluorites, after rinsing with water. The dried grains were fixed on stainless steel discus using 1 mm silicon oil stamps as adhesive (ca. 50 grains per aliquot).

Measurements were done on a Freiberg Instruments Lexsyg Smart device [82] using the single aliquot regenerative dose (SAR) protocol [71], with preheating at 230°C for 10 s as identified appropriate in dose recovery tests. The OSL decay curves are quite bright, dominated by the fast component and show no problem with regard to feldspar contamination. 40 aliquots were measured per sample but some had to be discarded due to low signal output. The D_e distributions (S11 Fig) show a distinct peak at the lower edge of the distribution with some outliers towards higher values for samples AJR1 and AJR2. Sample AJR3 has a quite narrow and Gaussian-shaped distribution, with an overdispersion (od.) of 0.15. While this sample is considered to comprise only well-bleached grains, the other two samples presumably contain aliquots in which the OSL signal was incompletely reset at the time of deposition. The samples also show higher overdispersion values (AJR2 = 0.28, AJR3 = 0.58). To calculate mean D_e, the Central Age Model (CAM) was used for AJR3 and the Minimum Age Model (MAM) for AJR1 and AJR2 (cf. [72]). The overdisperion observed for the apparently well-bleached sample AJR3 was used as input parameter (sigma_b) in the MAM.

Samples for dose rate measurements (c. 200 g) were dried, ground and measured at VKTA Rossendorf e.V. for the concentration of K, Th and U by high-resolution gamma spectrometry (cf. [73]). The comparison of the activities determined for U-238 and Ra-226 show no indication for the presence of radioactive disequilibrium in any of the samples investigated. Water content was assumed at 3±3% considering the mainly arid conditions in the region. Dose rates and ages were calculated using

ADELEv2017 ([74]; add-ideas.de), taking into account longitude, latitude and sample depth for cosmic dose rate [83]. The dosimetric data and ages are compiled in Table S4.

References (continuing after main manuscript's references)

75. Hilbert YH, Crassard R, Charloux G, Loreto R. Nubian technology in northern Arabia: Impact on interregional variability of Middle Palaeolithic industries. Quat. Int. 2017;435: 77-93.

76. Charloux G. Rythmes et modalités du peuplement d'une oasis du nord-ouest de l'Arabie. Sept campagnes (2010–2017) sur le site de Dûmat al-Jandal. Comptes rendus des séances de l'Académie des Inscriptions et Belles Lettres. 2018;2018(I): 11-46.

77. Crassard R, Hilbert YH. Bidirectional blade technology on naviform cores from northern Arabia: New evidence of Arabian-Levantine interactions in the Neolithic. Arabian Archaeology and Epigraphy. 2020;31(1): 93-104.

78. Hilbert YH, Crassard R. Middle and Late Pleistocene lithic technology from the region of Dûmat al-Jandal, northern Saudi Arabia. In: Bretzke K, Crassard R, Hilbert YH, editors. Stone Tools of Prehistoric Arabia. Oxford: Archaeopress; 2020. pp. 27-42.

79. Macholdt DS, Jochum KP, Al-Amri A, Andreae MO. Rock varnish on petroglyphs from the Hima region, southwestern Saudi Arabia: Chemical composition, growth rates, and tentative ages. The Holocene. 2019;29(8): 1377-1395..

80. Andreae MO, Al-Amri A, Andreae CM, Guagnin M, Jochum KP, Stoll B, Weis U. Archaeometric studies on the petroglyphs and rock varnish at Kilwa and Sakaka, northern Saudi Arabia. Arabian Archaeology and Epigraphy. 2020;31(2): 219-244.

81. Dorn RI, Dragovich D. Interpretation of Rock Varnish in Australia: case studies from the arid zone. Australian Geographer. 1990;21(1): 18-32.

82. Richter D, Richter A, Dornich K. Lexsyg smart – a luminescence detection system for dosimetry, material research and dating application. Geochronometria. 2015;42: 202-209.

83. Prescott JR, Hutton JT. Cosmic ray contributions to dose rates for luminescence and ESR dating: Large depths and long-term time variations. Radiation Measurements. 1994;23(2-3): 497-500.
