# Supplementary figures and images for: The oldest plans to scale of humanmade mega-structures
Source: PLoS One. 2023 May 17;18(5):e0277927. doi: 10.1371/journal.pone.0277927 (PMC10191280; doi:10.1371/journal.pone.0277927)

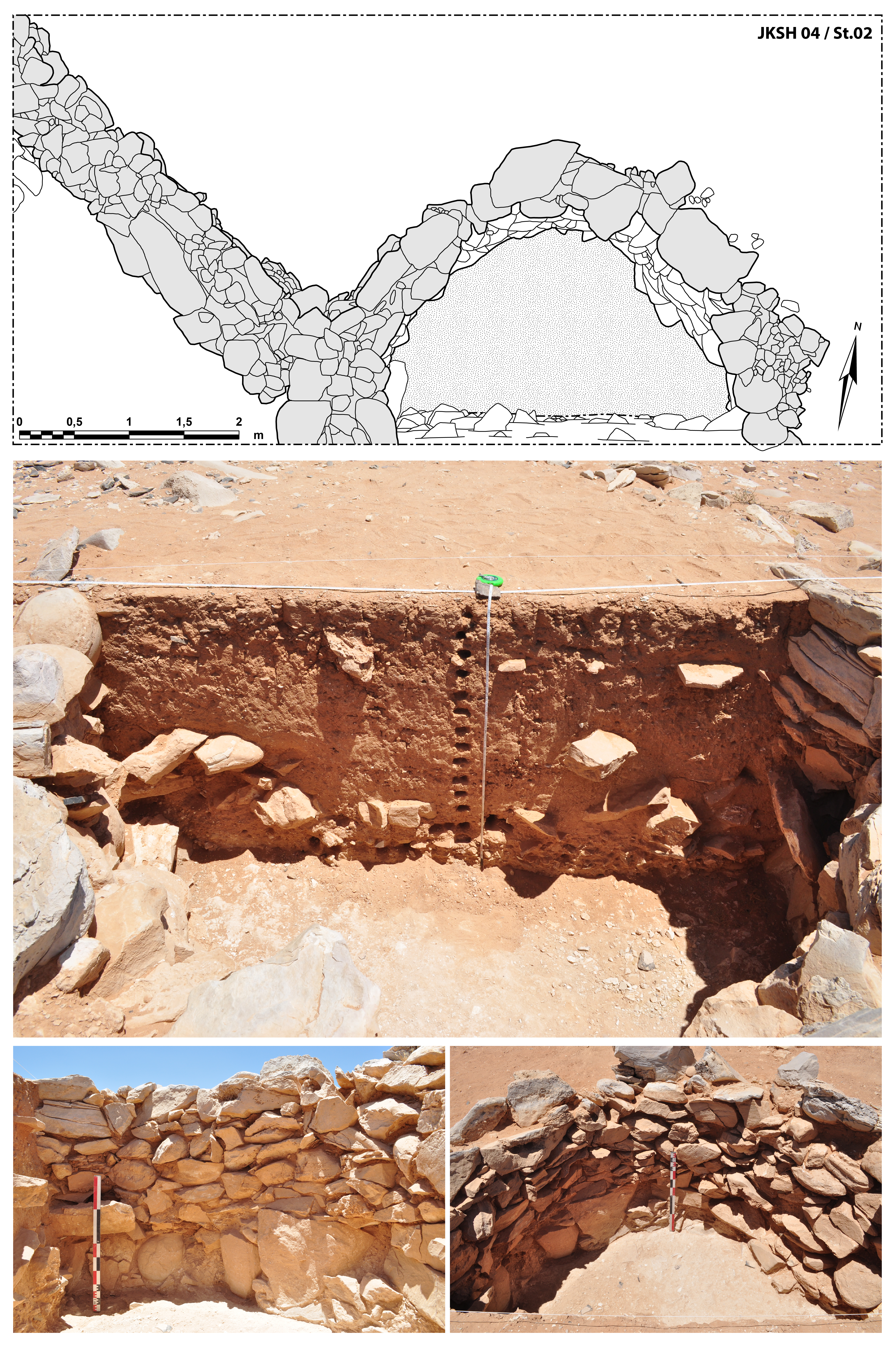

Supplement: S1 Fig — Top plan and different views of the excavation of pit trap St.02. (JPG) [file pone.0277927.s002.jpg]

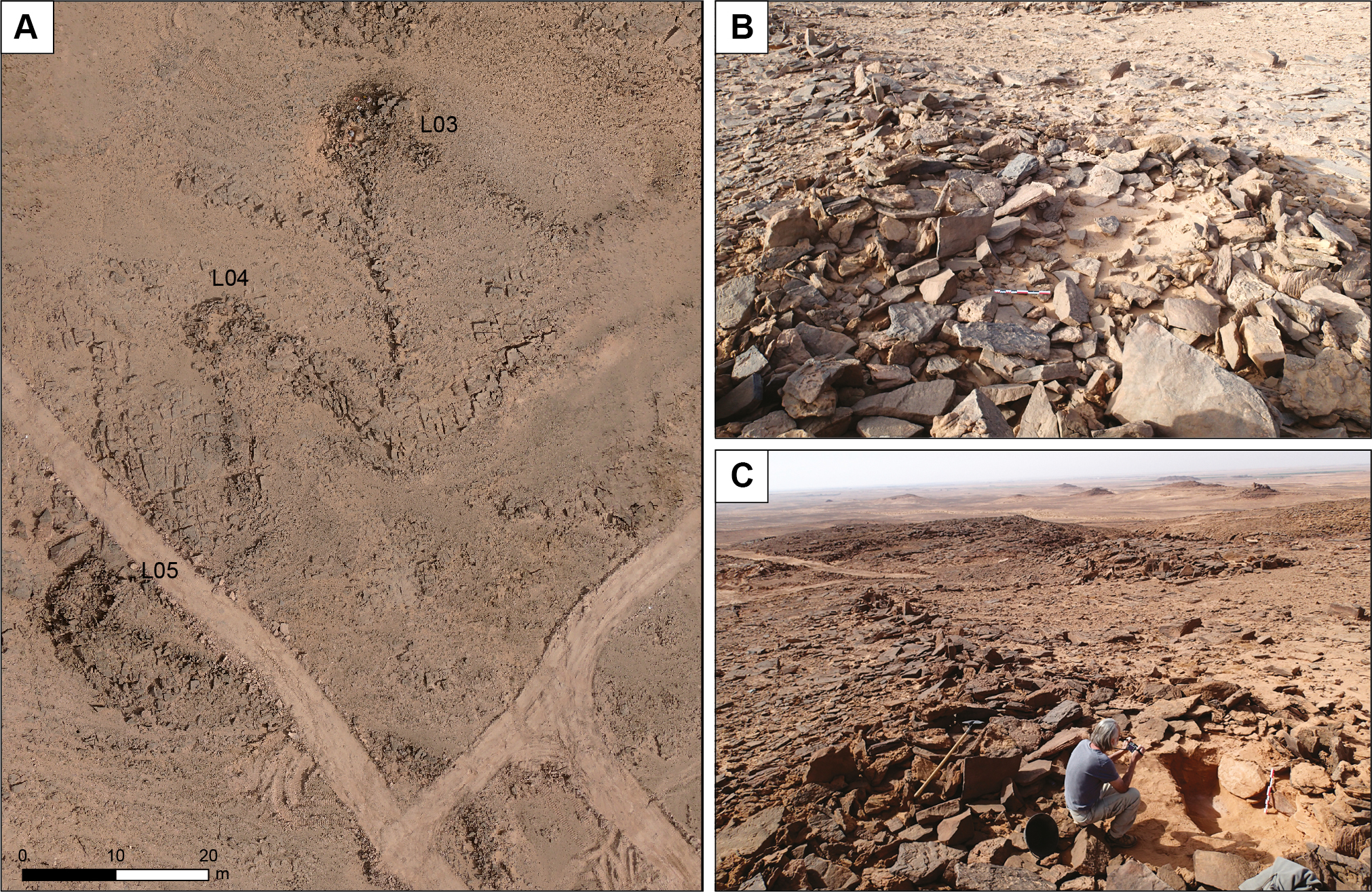

Supplement: S2 Fig — (A) Aerial view of pit-traps L03 (archaeological excavation), L04 and L05. (B) L03 before excavation. (C) L03 after excavation. (JPG) [file pone.0277927.s003.jpg]

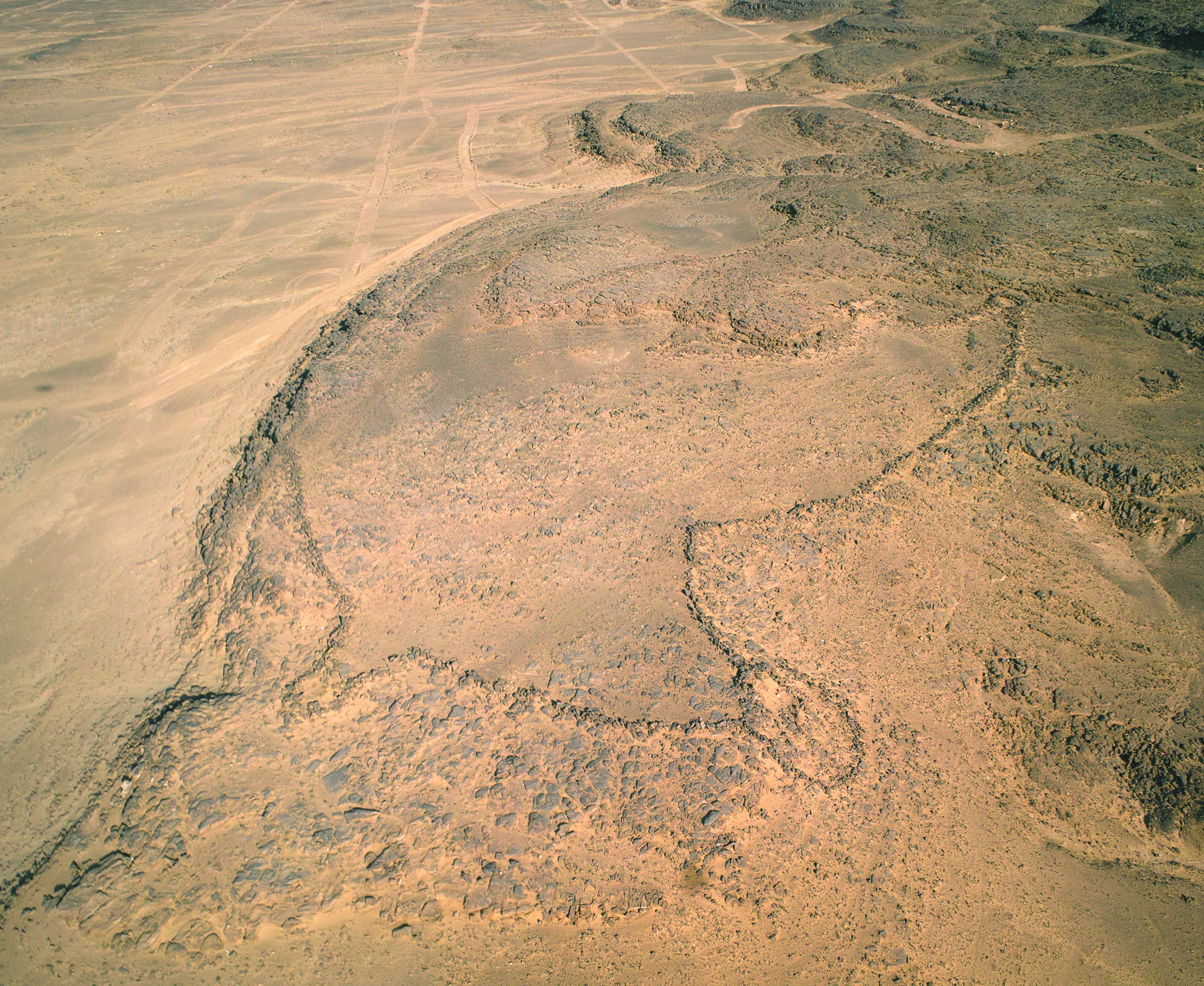

Supplement: S3 Fig — Aerial oblique view of the enclosure. (TIF) [file pone.0277927.s004.tif]

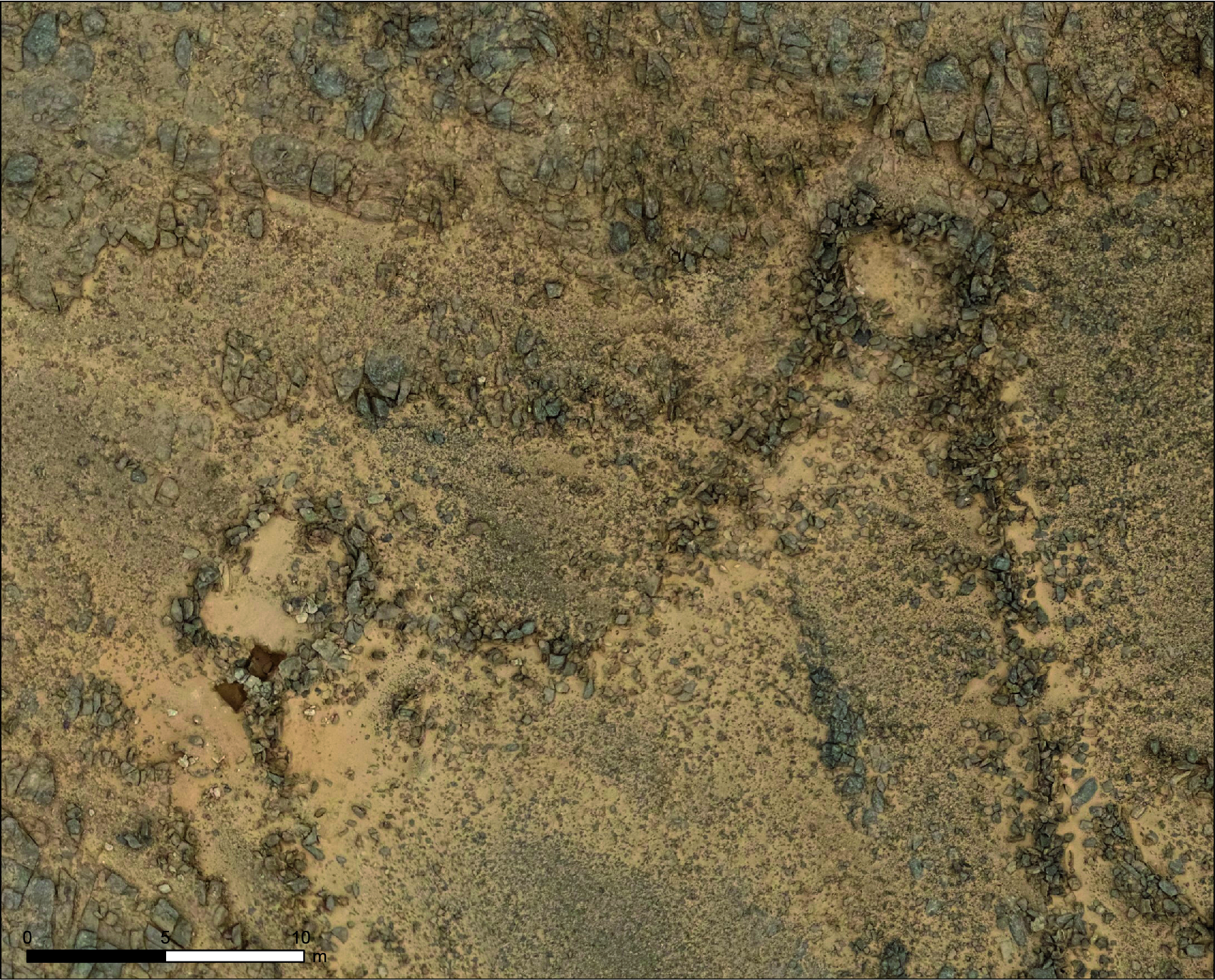

Supplement: S4 Fig — Aerial view of pit-traps L01 (archaeological excavation, on the left) and L02 (on the right). (JPG) [file pone.0277927.s005.jpg]

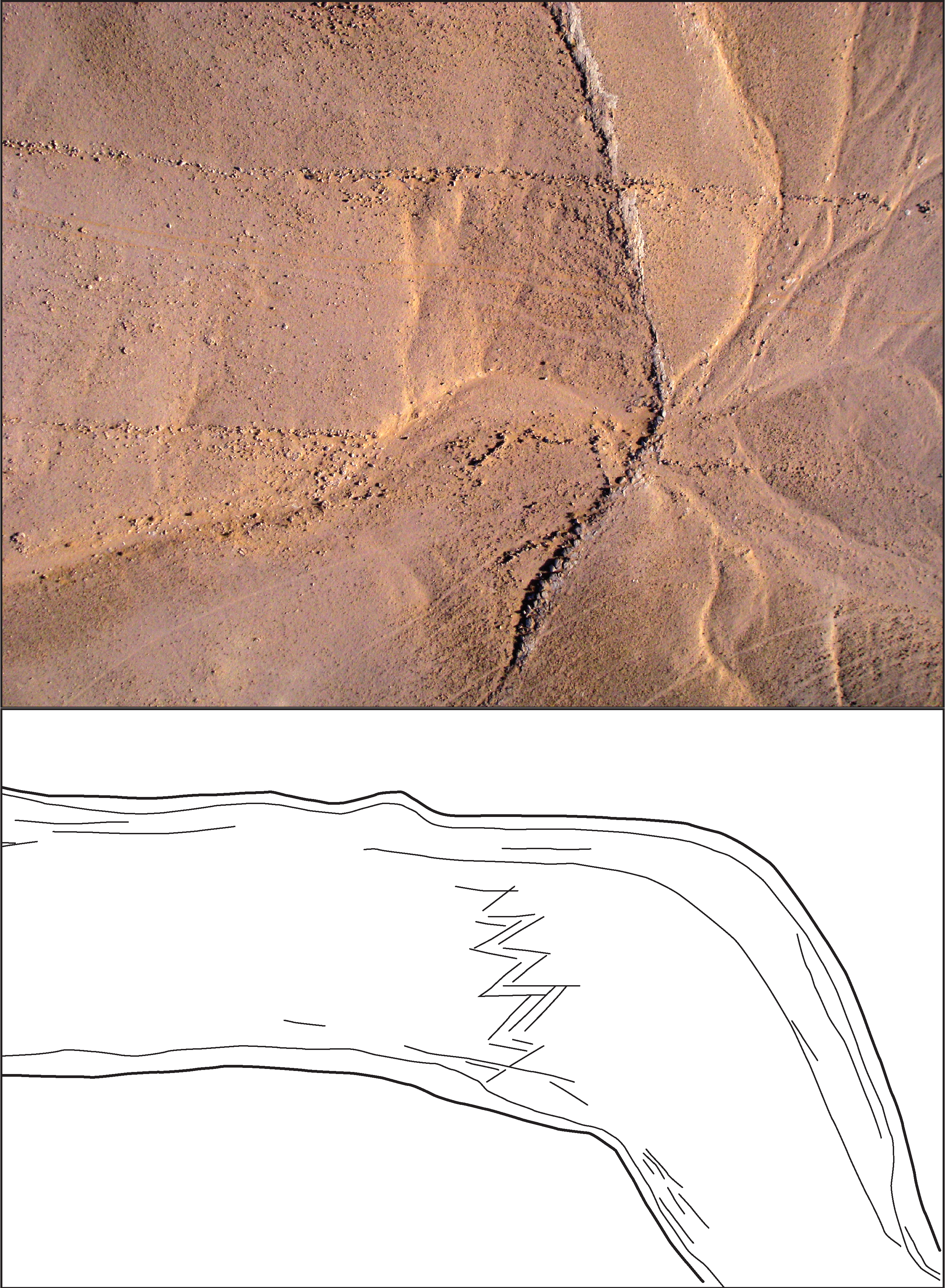

Supplement: S6 Fig — The detail of the chevron pattern (bottom) is compared to the slope break seen in the topography (here at kite JKSH 04, top). (JPG) [file pone.0277927.s007.jpg]

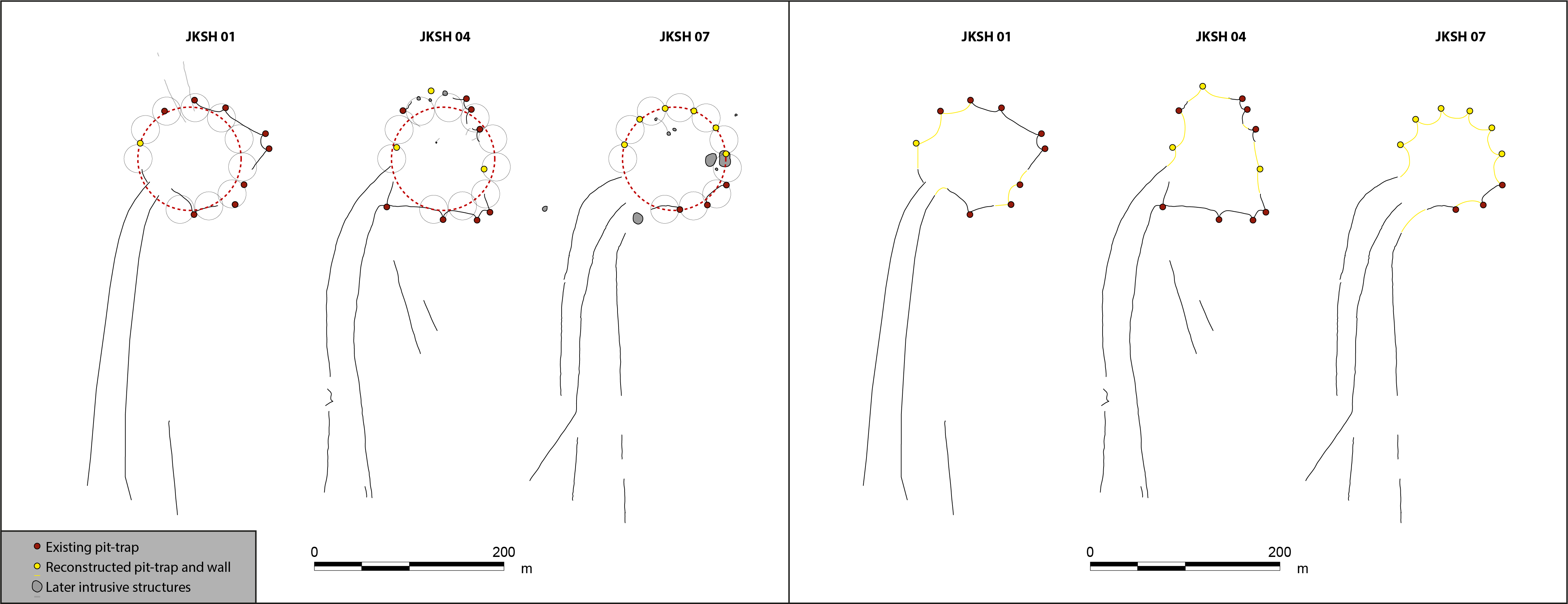

Supplement: S7 Fig — The reconstruction is based on the structural remains preserved in the field to estimate missing pit-traps around the enclosure’s perimeter. (JPG) [file pone.0277927.s008.jpg]

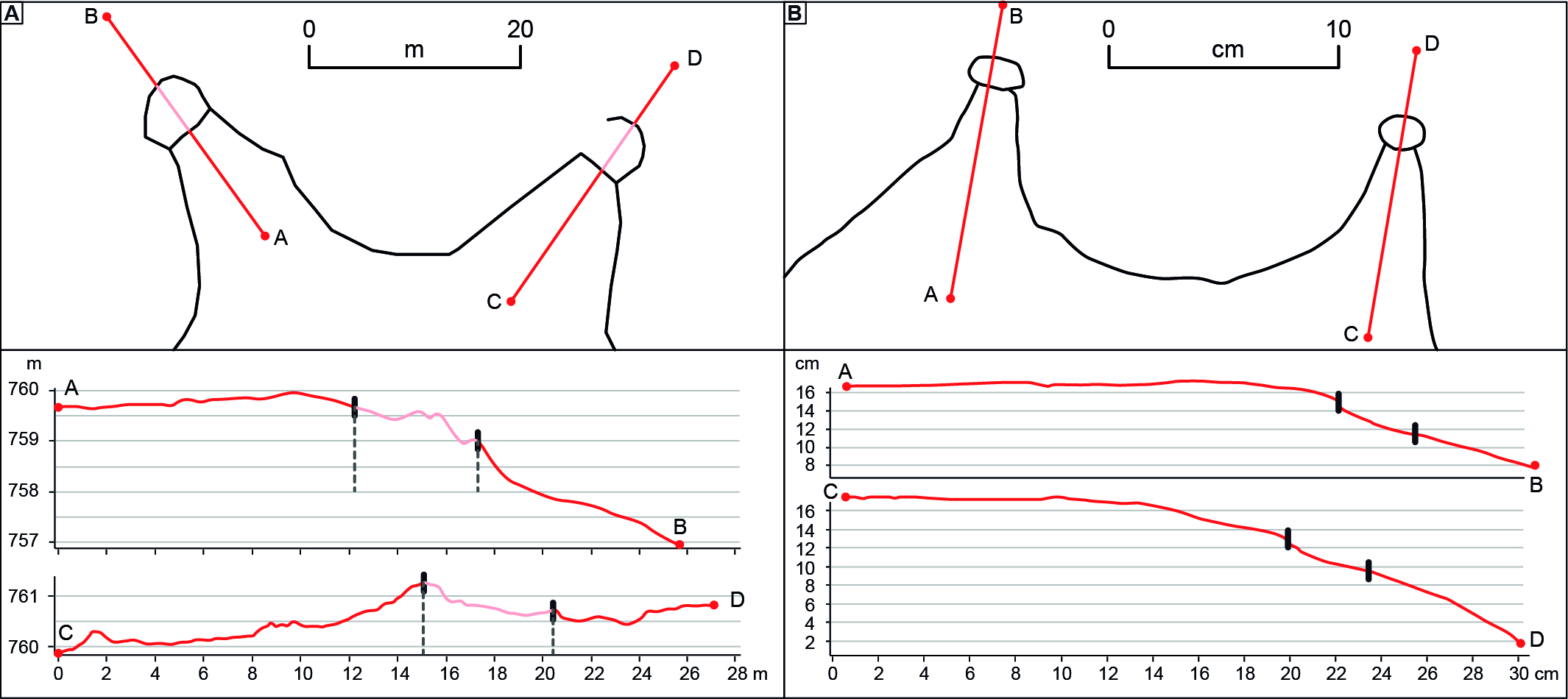

Supplement: S8 Fig — (A) Top-view and profiles of the southern part of desert kite AB135. (B) Top-view and profiles of the southern part of the engraving. (JPG) [file pone.0277927.s009.jpg]

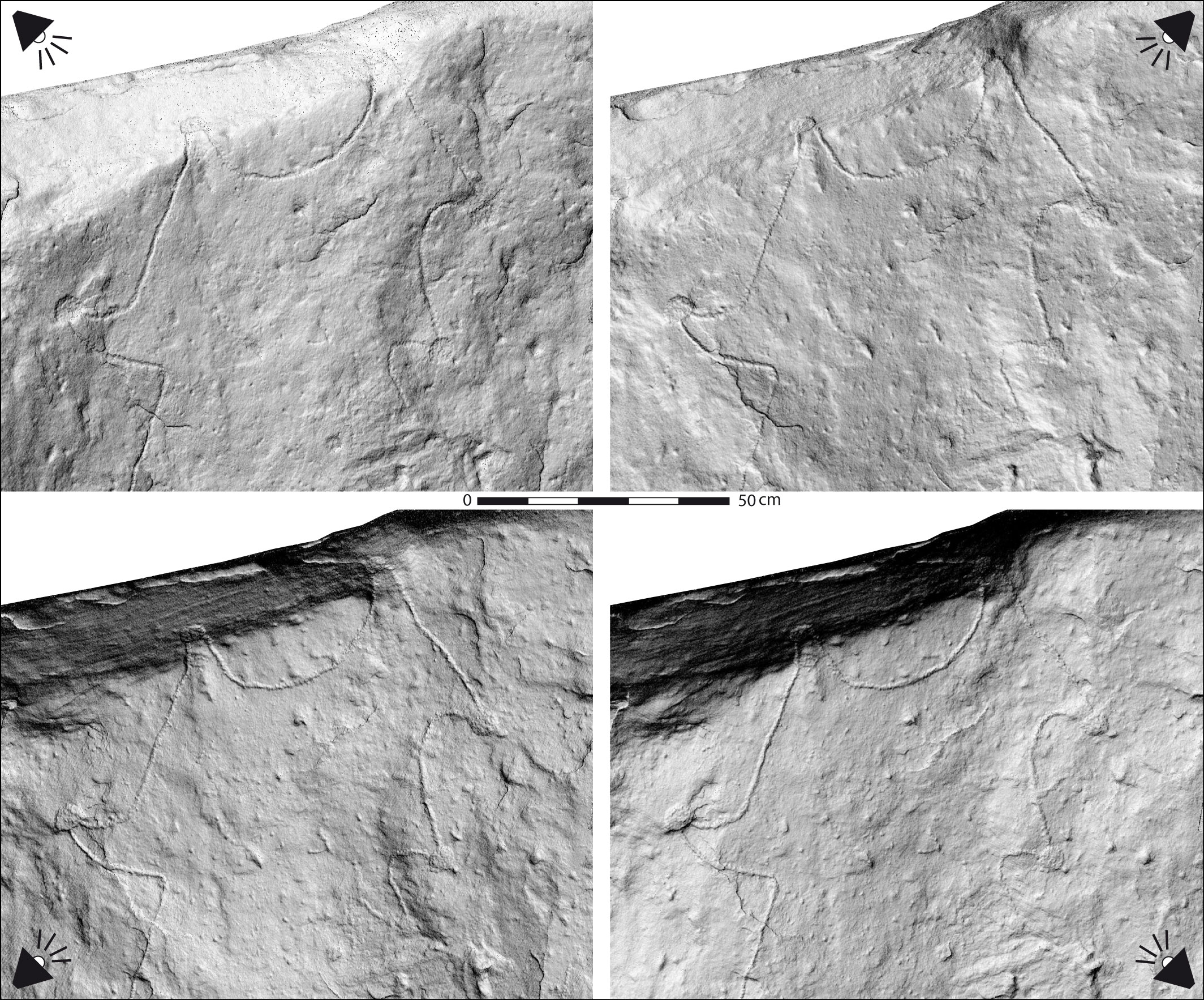

Supplement: S9 Fig — From the DTM, in ArcGIS, we produced a shaded image, showing black and white visualizations with low-angled light. It was made through four lighting angles (45°, 135°, 225°, 315°), revealing all engraved designs, even the most tenuous ones. (JPG) [file pone.0277927.s010.jpg]

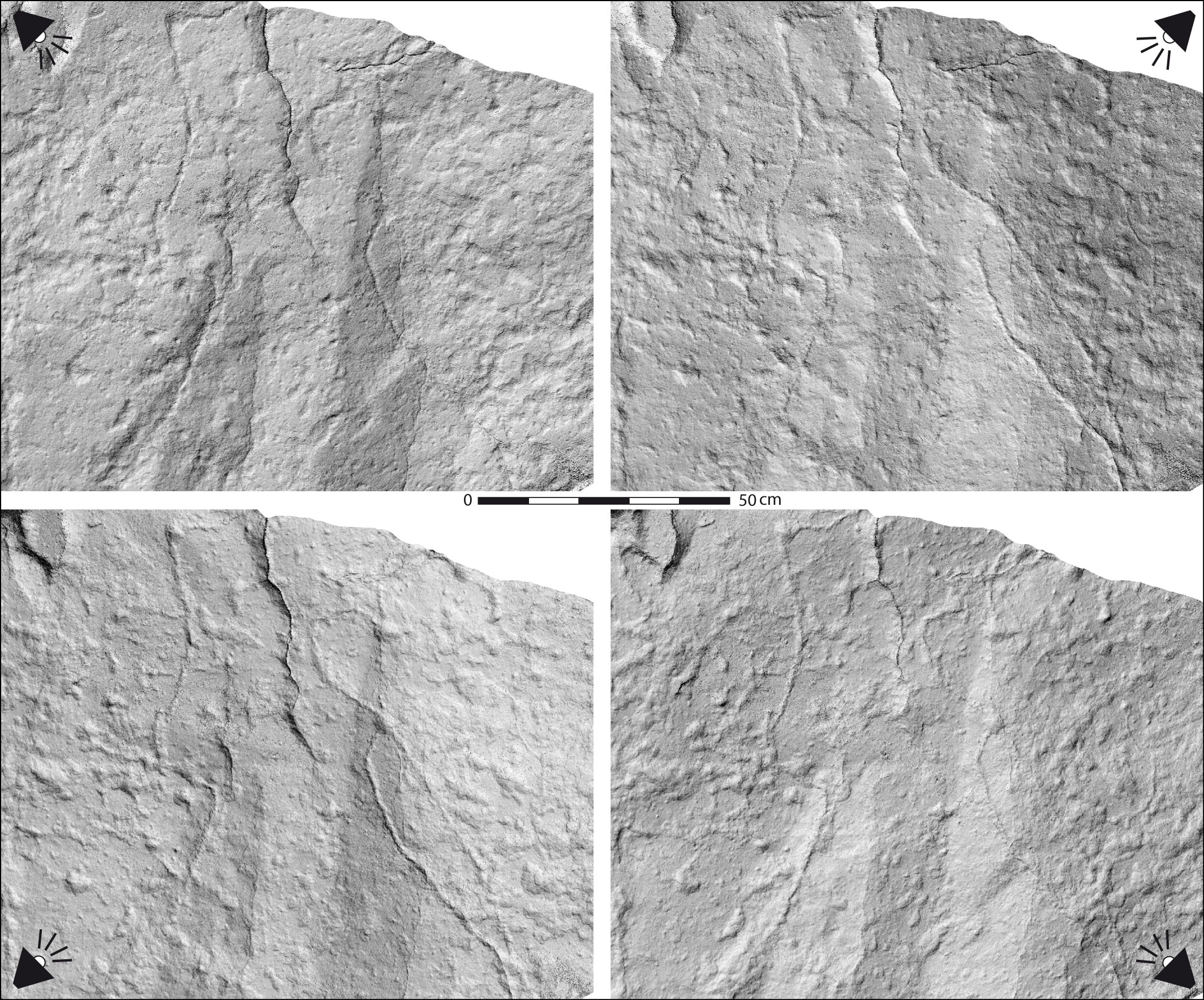

Supplement: S10 Fig — From the DTM, in ArcGIS, we produced a shaded image, showing black and white visualizations with low-angled light. It was made through four lighting angles (45°, 135°, 225°, 315°), revealing all engraved designs, even the most tenuous ones. (JPG) [file pone.0277927.s011.jpg]

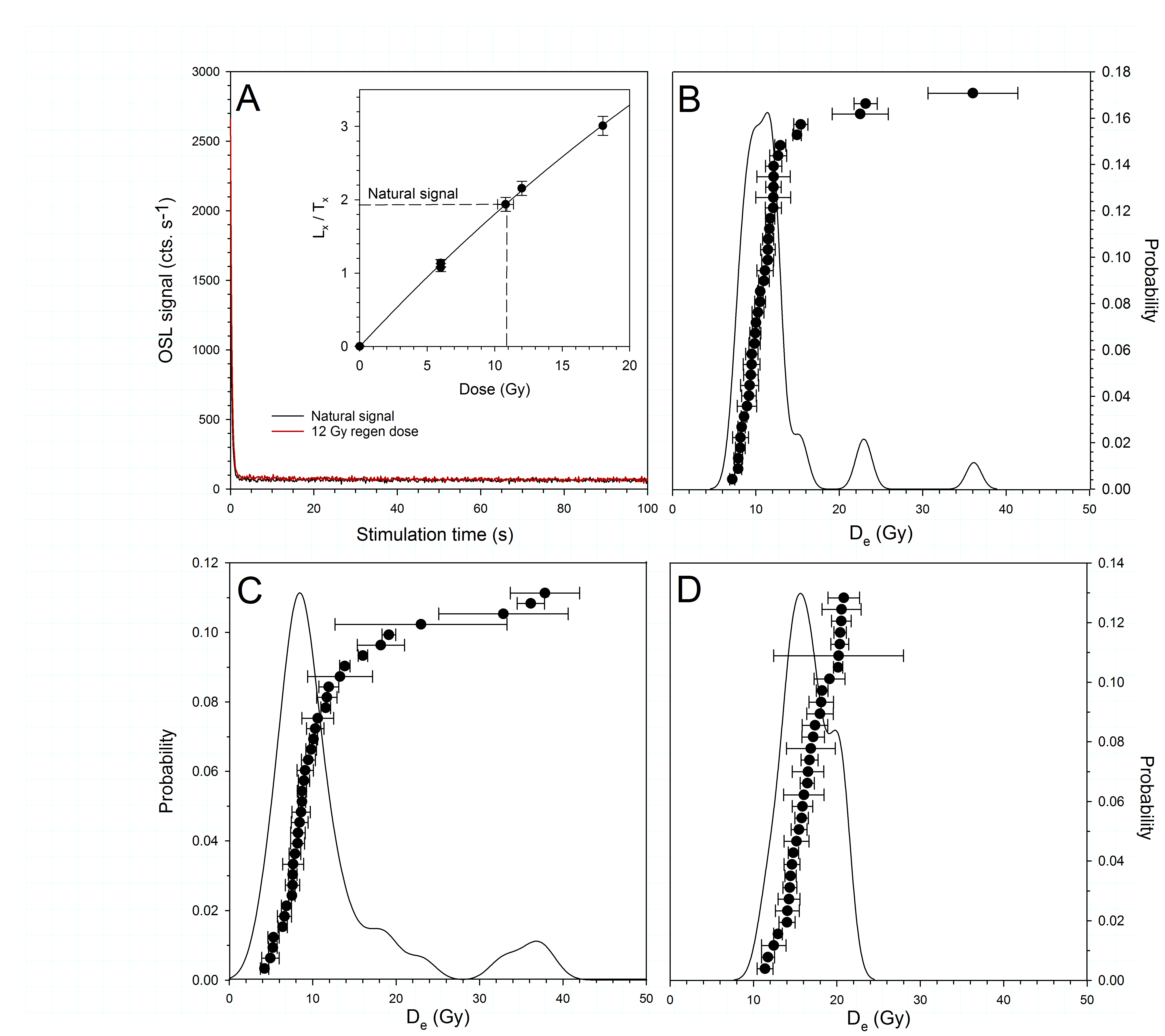

Supplement: S11 Fig — (A) Natural and laboratory irradiated OSL signals show almost identical decay shape with a dominance of the fast component (exemplified for AJR1). Inset: De values are in the close-to-linear range of signal growth. (B), (C), and (D) De distributions plots with density probability functions for samples AJR1-3 reveal a distinct peak in De values but some values at the higher edge that are interpreted to represent incomplete resetting of the OSL signal in quartz grains of some of the aliquots. (JPG) [file pone.0277927.s012.jpg]

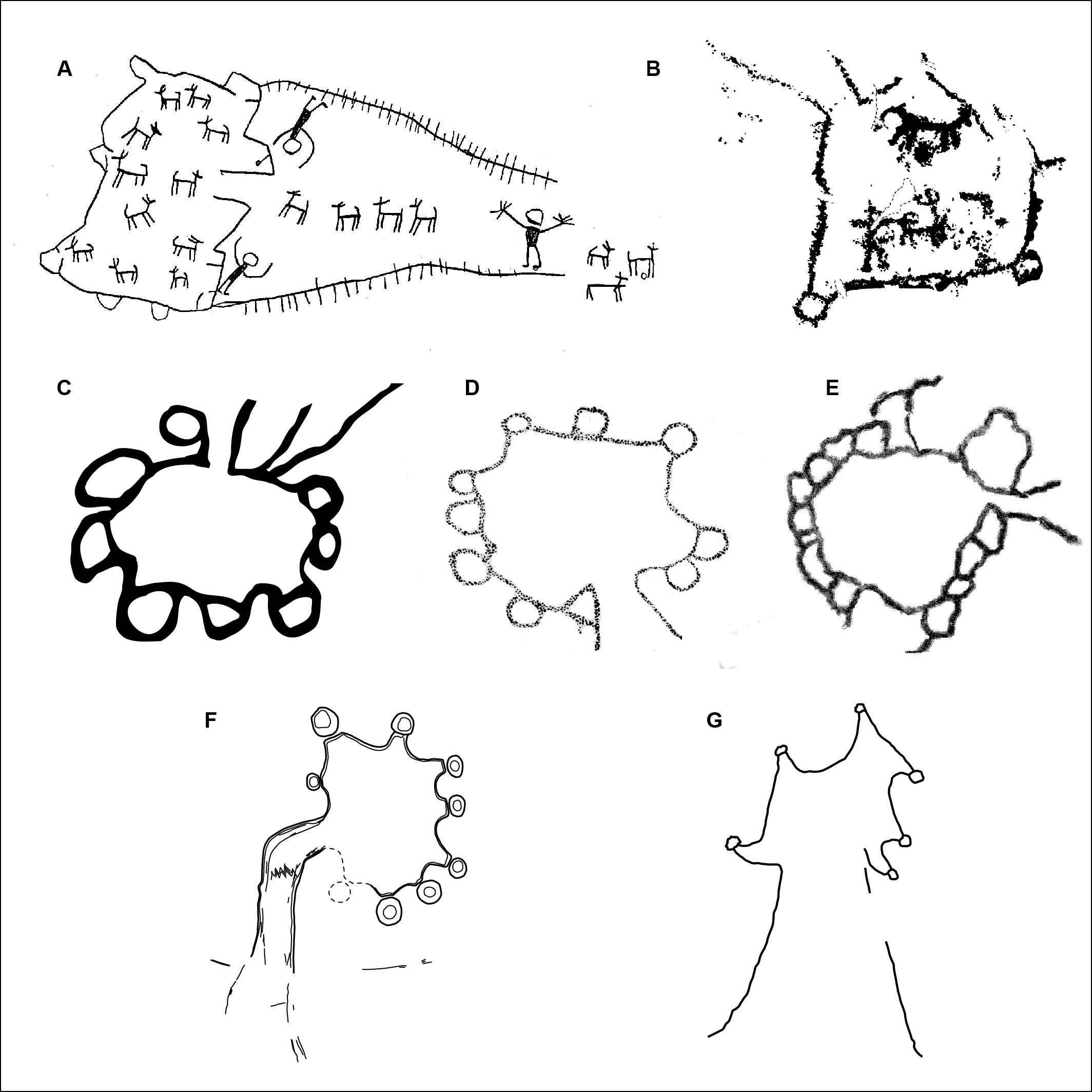

Supplement: S12 Fig — (A) From cairn of Hani’ site, Syria; redrawn from [42]:fig. 8. (B) From Khishâm-2 site, rock В 37, Syria; redrawn from [48]: fig. 3. (C) From Wisad Pools, Jordan; redrawn from [46]: fig. 44. (D) From Azraq Basin, Jordan; redrawn from [43]: fig. 3(6). (E) From Wisad Pools, Jordan; redrawn from [47]: fig. 8. (F) and (G) are the kite engravings described in the present study, respectively from Jibal al-Khashabiyeh and Jebel az-Zilliyat, represented in this figure to be directly compared with the previously known ones. (JPG) [file pone.0277927.s013.jpg]
