# Supplementary material for: The oldest plans to scale of humanmade mega-structures
Source: PLoS One. 2023 May 17;18(5):e0277927. doi: 10.1371/journal.pone.0277927 (PMC10191280; doi:10.1371/journal.pone.0277927)
Supplement: S1 Table — (PDF) [file pone.0277927.s014.pdf]

Supporting information

The oldest plans to scale of manmade mega-structures

Rémy Crassard, Wael Abu-Azizeh, Olivier Barge, Jacques Élie Brochier, Frank Preusser,
Hamida Seba, Abd Errahmane Kiouche, Emmanuelle Régagnon, Juan Antonio Sánchez Priego, Thamer Almalki,
Mohammad Tarawneh

S1 Table. Corresponding kite numbers between the scientific teams and GPS location of the Jibal al-Khashabiyeh desert kites.

Jibal al-Khashabiyeh project, site #	Globalkites Project inventory, kite #	Elevation (m asl)	Latitude	Longitude
JKSH 01	JD1088	969	30.154N	37.021E
JKSH 02	JD1089	969	30.165N	37.011E
JKSH 03	JD1090	977	30.194N	37.002E
JKSH 04	JD1091	971	30.214N	36.978E
JKSH 05	JD1092	970	30.229N	36.991E
JKSH 06	JD1093	970	30.252N	37.001E
JKSH 07	JD1094	976	30.278N	36.953E
JKSH 08	JD1095	965	30.296N	36.951E
