# Supplementary material for: The oldest plans to scale of humanmade mega-structures
Source: PLoS One. 2023 May 17;18(5):e0277927. doi: 10.1371/journal.pone.0277927 (PMC10191280; doi:10.1371/journal.pone.0277927)
Supplement: S2 Table — (PDF) [file pone.0277927.s015.pdf]

Supporting information

The oldest plans to scale of manmade mega-structures

Rémy Crassard, Wael Abu-Azizeh, Olivier Barge, Jacques Élie Brochier, Frank Preusser,
Hamida Seba, Abd Errahmane Kiouche, Emmanuelle Régagnon, Juan Antonio Sánchez Priego, Thamer Almalki, Mohammad Tarawneh

S2 Table. Corresponding kite numbers between the scientific teams and GPS location of the Jebel az-Zilliyat desert kites.

Dumat al-Jandal project site #	Globalkites Project inventory, kite #	Elevation (m asl)	Latitude	Longitude
DAJ137	AB135	766	29.910N	39.712E
DAJ138	AB136	773	29.910N	39.715E
DAJ139	AB547	748	29.926E	39.683E
DAJ140	AB549	741	29.924N	39.680E
