# Supplementary material for: The oldest plans to scale of humanmade mega-structures
Source: PLoS One. 2023 May 17;18(5):e0277927. doi: 10.1371/journal.pone.0277927 (PMC10191280; doi:10.1371/journal.pone.0277927)
Supplement: S3 Table — Dates were calibrated with Chronomodel v.2 [69] using IntCal20 atmospheric calibration curve [70]. Md, Q1 and Q3 refers to the median, first and third quartile of the distribution of calendar ages. Bold characters underline the earliest date obtained for each kite (i.e. the radiocarbon terminus ante quem of the construction). All calendar dates are rounded to the nearest ten. * Unreliable sample (0.24 mg of carbon only, very large standard error): not considered. (PDF) [file pone.0277927.s016.pdf]

Supporting information

The oldest plans to scale of manmade mega-structures

Rémy Crassard, Wael Abu-Azizeh, Olivier Barge, Jacques Élie Brochier, Frank Preusser,
Hamida Seba, Abd Errahmane Kiouche, Emmanuelle Régagnon, Juan Antonio Sánchez Priego, Thamer Almalki, Mohammad Tarawneh

S3 Table. Radiocarbon analysis results from Jibal al-Khashabiyeh, Jordan, and Jebel az-Zilliyat, Saudi Arabia. Dates were calibrated with Chronomodel v.2 (*69*) using IntCal20 atmospheric calibration curve (*70*). Md, Q1 and Q3 refers to the median, first and third quartile of the distribution of calendar ages. Bold characters underline the earliest date obtained for each kite (*i.e.* the radiocarbon *terminus ante quem* of the construction). All calendar dates are rounded to the nearest ten. * Unreliable sample (0.24 mg of carbon only, very large standard error): not considered.

						**calendar ages BC**		
**sample id.**	**Lab id.**	**Depth (cm)**	**material**	**age BP**	**error**	**95% ranges**	**Md (Q1, Q3)**	**70% ranges**
JKSH 01 S1	AA106905	70	charcoal	7580	38	[6500 - 6490] 0,8%<br>[6480 - 6380] 94,2%	6440 (6450, 6420)	[6460 - 6420] 70%
	AA106906	130	charcoal	7985	37	[7050 - 6770] 90,8%<br>[6760 - 6750] 1,1%<br>[6720 - 6700] 3,2%	6910 (6980, 6840)	[7040 -6980] 22,2%<br>[6970 - 6910] 25,6%<br>[6890 - 6830] 22,3%
JKSH 01 S10	AA106907	120	charcoal	8100	43	[7310 - 7280] 2,2%<br>[7250 - 7230] 1,8%<br>[7190 - 7030] 84%<br>[6970 - 6950] 1,7%<br>[6930 - 6910] 1,4%<br>[6880 -6830] 4,0%	7080 (7130, 7060)	[7170 - 7160] 4,6%<br>[7140 - 7100] 28,1%<br>[7080 - 7040] 37,5%
	AA106908	150	charcoal	8051	49	[7170 - 7160] 0,4%<br>[7140 - 7100] 5,3%<br>[7080 - 6770] 88,8%<br>[6710 - 6710] 0,6%	6960 (7050, 6870)	[7070 - 7020] 25,9%<br>[7010 - 6990] 4,7%<br>[6970 - 6940] 10,2%<br>[6940 - 6910] 9,5%<br>[6880 - 6830] 19,9%
	AA106909	170	charcoal	**8154**	**31**	**[7310 - 7270] 6,8%**<br>**[7250 - 7230] 5,8%**<br>**[7190 - 7060] 82,5%**	**7130 (7170, 7100)**	**[7170 - 7090] 57,3%**<br>**[7090 - 7070] 12,9%**
JKSH 04 S1	AA106910	65	charcoal	**7029**	**29**	**[5980 - 5840] 95%**	**5920 (5960, 5890)**	**[5980 - 5940] 33,4%**<br>**[5920 - 5890] 33,6%**<br>**[5860 - 5850] 3,1%**
	AA106911*	150	charcoal	9010	140	[8550 - 7740] 95%	8160 (8290, 8000)	[8120 - 8410] 1,3%<br>[8390 - 8370] 2,0%<br>[8350 - 7940] 66,8%
JKSH F15	AA106914	–	charcoal	8016	29	[7060 - 6820] 94,9%<br>[6780 - 6780] 0,2%	6930 (7000, 6870)	[7050 - 7030] 13,7%<br>[7010 - 7000] 3,4%<br>[6970 - 6910] 11,8%<br>[6880 - 6830] 27,3%
AB 135 LO3	Poz 107811	45	*Arnebia* nutlets	6760	40	[5730 - 5620] 93,8%<br>[5580 - 5570] 1,4%	5670 (5690, 5640)	[5710 - 5690] 23,5%<br>[5670 - 5630] 47,3%
AB 549 LO1	Poz 116104	49	*Arnebia* nutlets	3300	40	[1680 -1650] 4,5%<br>[1640 - 1500] 89,1%<br>[1470 - 1460] 1,4%	1570 (1600, 1540)	[1610 - 1530] 64,8%<br>[1530 - 1520] 5,7%
