# Supplementary material for: The oldest plans to scale of humanmade mega-structures
Source: PLoS One. 2023 May 17;18(5):e0277927. doi: 10.1371/journal.pone.0277927 (PMC10191280; doi:10.1371/journal.pone.0277927)
Supplement: S4 Table — AB135 stands for DAJ137 kite, and AB549 stands for DAJ140 kite. (PDF) [file pone.0277927.s017.pdf]

Supporting information

The oldest plans to scale of manmade mega-structures

Rémy Crassard, Wael Abu-Azizeh, Olivier Barge, Jacques Élie Brochier, Frank Preusser, Hamida Seba, Abd Errahmane Kiouche, Emmanuelle Régagnon, Juan Antonio Sánchez Priego, Thamer Almalki, Mohammad Tarawneh

S4 Table. Summarized OSL dating data from Jebel az-Zilliyat, Saudi Arabia. AB135 stands for DAJ137 kite, and AB549 stands for DAJ140 kite.

Lab code	Sample	Depth (cm)	K (%)	Th (ppm)	U (ppm)	D (Gy ka^{-1})	n	od	D_e (Gy)	Age (a)	Age (BC)
AJR1	AB549L1-OSL1	65	0.30 ± 0.03	5.56 ± 0.35	1.25 ± 0.22	1.20 ± 0.05	33	0.28	9.67 ± 0.28	8040 ± 430	6455-5595
AJR2	AB549L1-OSL2	65	0.29 ± 0.03	4.96 ± 0.33	1.36 ± 0.25	1.17 ± 0.06	38	0.59	8.74 ± 0.32	7480 ± 460	5925-5005
AJR3	AB135L03-OSL1	30	0.67 ± 0.06	8.96 ± 0.57	2.53 ± 0.36	2.11 ± 0.09	38	0.15	16.23± 0.48	7690 ± 400	6075-5275
