# Supplementary material for: The oldest plans to scale of humanmade mega-structures
Source: PLoS One. 2023 May 17;18(5):e0277927. doi: 10.1371/journal.pone.0277927 (PMC10191280; doi:10.1371/journal.pone.0277927)
Supplement: S5 Table — (PDF) [file pone.0277927.s018.pdf]

Supporting information

The oldest plans to scale of manmade mega-structures

Rémy Crassard, Wael Abu-Azizeh, Olivier Barge, Jacques Élie Brochier, Frank Preusser, Hamida Seba, Abd Errahmane Kiouche, Emmanuelle Régagnon, Juan Antonio Sánchez Priego, Thamer Almalki, Mohammad Tarawneh

S5 Table. Similarity scores obtained when comparing kites to the engraving found at Jebel az-Zilliyat, Saudi Arabia.

Archaeological kite	Similarity score to the engraving found at Jebel az-Zilliyat	Distance (km) between archaeological kite and the engraving
AB547	74.04%	1.73
AB549	80.80%	2.03
**AB135**	**81.43%**	**2.30**
AB136	68.73%	2.42
AB621	37.64%	22.43
AB550	61.99%	26.56
AB573	27.84%	63.71
AB558	50.75%	77.08
AB543	59.12%	190.66
AB178	63.28%	226.95
AB121	55.17%	249.25
JD159	34.71%	260.82
AB441	46.64%	266.04
JD156	40.70%	269.52
AB438	72.98%	275.30
JD180	61.36%	281.06
JD174	59.21%	287.81
JD136	50.30%	302.53
JD124	53.47%	315.82
JD99	43.63%	322.66
JD919	72.40%	325.04
JD255	67.53%	340.71
JD208	59.49%	341.14
JD273	51.91%	344.68
JD482	34.84%	352.57
JD11	57.28%	358.72
JD305	57.73%	360.61
JD494	36.58%	362.14
SY228	59.56%	397.16
SY1627	49.75%	400.30
SY55	63.29%	403.27
SY1506	59.90%	404.77
SY223	51.04%	406.00
SY1274	52.01%	411.64
SY1270	38.25%	418.49
AB173	61.52%	422.44
SY171	42.70%	426.86

Supporting information

The oldest plans to scale of manmade mega-structures

Rémy Crassard, Wael Abu-Azizeh, Olivier Barge, Jacques Élie Brochier, Frank Preusser,
Hamida Seba, Abd Errahmane Kiouche, Emmanuelle Régagnon, Juan Antonio Sánchez Priego, Thamer Almalki, Mohammad Tarawneh

AB111	48.67%	441.09
AB58	60.40%	460.02
AB223	52.43%	460.26
AB210	71.06%	461.13
AB277	41.66%	462.66
AB377	62.18%	464.85
AB247	62.23%	479.19
AB298	45.12%	484.01
SY464	56.42%	485.49
SY1180	61.10%	510.97
SY703	45.56%	526.01
SY274	53.37%	533.79
SY101	55.84%	537.22
SY107	43.89%	539.59
SY1059	69.52%	551.13
SY729	73.39%	554.58
SY1014	50.25%	577.14
SY1043	46.15%	588.55
SY714	63.91%	744.13
TK16	47.52%	786.47
AM28	63.92%	1192.54
AM36	30.61%	1208.15
KZ2	26.83%	1994.81
KZ355	45.62%	2160.24
KZ344	43.29%	2238.25
KZ345	42.91%	2248.91
KZ111	38.50%	2355.15
KZ12	43.51%	2358.27
KZ122	44.46%	2371.71
