# Supplementary material for: The oldest plans to scale of humanmade mega-structures
Source: PLoS One. 2023 May 17;18(5):e0277927. doi: 10.1371/journal.pone.0277927 (PMC10191280; doi:10.1371/journal.pone.0277927)
Supplement: S6 Table — (PDF) [file pone.0277927.s019.pdf]

**Supporting information**

**The oldest plans to scale of manmade mega-structures**

Rémy Crassard, Wael Abu-Azizeh, Olivier Barge, Jacques Élie Brochier, Frank Preusser,
Hamida Seba, Abd Errahmane Kiouche, Emmanuelle Régagnon, Juan Antonio Sánchez Priego, Thamer Almalki, Mohammad Tarawneh

**S6 Table.** Similarity scores obtained when comparing kites to the engraving found at Jibal al-Khashabiyeh, Jordan.

| Archaeological kite | Similarity score to the engraving found at Jibal al-Khashabiyeh | Distance (km) between archaeological kite and the engraving |
|---|---|---|
| JKSH 07 | 73.52% | 1.428 |
| JKSH 04 | 60.62% | 8.696 |
| **JKSH 01** | **75.90%** | **16.311** |
| AB543 | 67.78% | 142.345 |
| AB438 | 66.93% | 149.055 |
| AB178 | 71.26% | 159.564 |
| AB441 | 68.15% | 172.834 |
| JD482 | 19.85% | 180.47 |
| AB121 | 62.85% | 183.224 |
| JD156 | 45.59% | 187.628 |
| JD159 | 48.63% | 191.733 |
| JD99 | 50.25% | 192.646 |
| JD273 | 61.67% | 194.261 |
| JD919 | 56.43% | 197.155 |
| JD208 | 35.11% | 199.939 |
| JD11 | 34.29% | 210.923 |
| JD180 | 70.16% | 212.087 |
| JD174 | 58.78% | 217.604 |
| JD136 | 51.89% | 219.295 |
| JD255 | 46.33% | 224.328 |
| JD494 | 25.15% | 224.378 |
| AB550 | 51.39% | 240.578 |
| AB573 | 11.46% | 240.649 |
| AB621 | 24.62% | 244.26 |
| JD124 | 55.56% | 247.617 |
| JD305 | 53.66% | 249.065 |
| SY1627 | 33.89% | 257.963 |
| AB549 | 58.51% | 262.933 |
| AB547 | 56.00% | 263.178 |
| AB135 | 59.47% | 266.226 |
| AB136 | 44.53% | 266.469 |
| SY223 | 35.58% | 271.397 |
| AB558 | 30.63% | 273.713 |
| SY228 | 67.88% | 276.48 |
| SY1506 | 56.75% | 288.618 |
| SY1274 | 48.41% | 290.3 |
| SY55 | 72.68% | 291.982 |

**Supporting information**

**The oldest plans to scale of manmade mega-structures**

Rémy Crassard, Wael Abu-Azizeh, Olivier Barge, Jacques Élie Brochier, Frank Preusser,
Hamida Seba, Abd Errahmane Kiouche, Emmanuelle Régagnon, Juan Antonio Sánchez Priego, Thamer Almalki, Mohammad Tarawneh

| | | |
|---|---|---|
| SY1270 | 20.38% | 294.638 |
| SY171 | 23.77% | 299.496 |
| SY464 | 33.19% | 402.036 |
| SY703 | 57.08% | 425.842 |
| SY101 | 60.69% | 431.268 |
| SY107 | 63.57% | 434.812 |
| SY729 | 54.28% | 451.019 |
| SY1180 | 44.87% | 456.675 |
| SY274 | 44.74% | 485.186 |
| AB173 | 58.64% | 520.354 |
| SY1059 | 47.54% | 521.174 |
| AB111 | 33.77% | 530.55 |
| AB277 | 51.63% | 538.71 |
| AB223 | 38.80% | 541.688 |
| AB58 | 49.64% | 544.749 |
| AB377 | 57.59% | 545.179 |
| AB210 | 60.45% | 545.467 |
| SY1014 | 74.53% | 560.103 |
| AB247 | 56.13% | 560.338 |
| SY1043 | 36.71% | 569.446 |
| AB298 | 40.99% | 571.673 |
| TK16 | 33.28% | 748.792 |
| SY714 | 63.85% | 764.437 |
| AM28 | 35.81% | 1239.828 |
| AM36 | 13.27% | 1254.303 |
| KZ2 | 15.20% | 2113.052 |
| KZ355 | 33.38% | 2262.339 |
| KZ344 | 26.96% | 2335.8 |
| KZ345 | 26.52% | 2347.347 |
| KZ111 | 21.34% | 2468.647 |
| KZ12 | 23.80% | 2473.088 |
| KZ122 | 23.85% | 2484.228 |
